# A plasmid DNA-launched SARS-CoV-2 reverse genetics system and coronavirus toolkit for COVID-19 research

**DOI:** 10.1371/journal.pbio.3001091

**Published:** 2021-02-25

**Authors:** Suzannah J. Rihn, Andres Merits, Siddharth Bakshi, Matthew L. Turnbull, Arthur Wickenhagen, Akira J. T. Alexander, Carla Baillie, Benjamin Brennan, Fiona Brown, Kirstyn Brunker, Steven R. Bryden, Kerry A. Burness, Stephen Carmichael, Sarah J. Cole, Vanessa M. Cowton, Paul Davies, Chris Davis, Giuditta De Lorenzo, Claire L. Donald, Mark Dorward, James I. Dunlop, Matthew Elliott, Mazigh Fares, Ana da Silva Filipe, Joseph R. Freitas, Wilhelm Furnon, Rommel J. Gestuveo, Anna Geyer, Daniel Giesel, Daniel M. Goldfarb, Nicola Goodman, Rory Gunson, C. James Hastie, Vanessa Herder, Joseph Hughes, Clare Johnson, Natasha Johnson, Alain Kohl, Karen Kerr, Hannah Leech, Laura Sandra Lello, Kathy Li, Gauthier Lieber, Xiang Liu, Rajendra Lingala, Colin Loney, Daniel Mair, Marion J. McElwee, Steven McFarlane, Jenna Nichols, Kyriaki Nomikou, Anne Orr, Richard J. Orton, Massimo Palmarini, Yasmin A. Parr, Rute Maria Pinto, Samantha Raggett, Elaine Reid, David L. Robertson, Jamie Royle, Natalia Cameron-Ruiz, James G. Shepherd, Katherine Smollett, Douglas G. Stewart, Meredith Stewart, Elena Sugrue, Agnieszka M. Szemiel, Aislynn Taggart, Emma C. Thomson, Lily Tong, Leah S. Torrie, Rachel Toth, Margus Varjak, Sainan Wang, Stuart G. Wilkinson, Paul G. Wyatt, Eva Zusinaite, Dario R. Alessi, Arvind H. Patel, Ali Zaid, Sam J. Wilson, Suresh Mahalingam

**Affiliations:** 1 MRC-University of Glasgow Centre for Virus Research (CVR), Glasgow, United Kingdom; 2 Institute of Technology, University of Tartu, Tartu, Estonia; 3 MRC Protein Phosphorylation and Ubiquitylation Unit, School of Life Sciences, University of Dundee, Dundee, United Kingdom; 4 Institute of Biodiversity, Animal Health and Comparative Medicine, University of Glasgow, Glasgow, United Kingdom; 5 Emerging Viruses, Inflammation and Therapeutics Group, Menzies Health Institute Queensland, Griffith University, Gold Coast, Queensland, Australia; 6 Division of Biological Sciences, College of Arts and Sciences, University of the Philippines Visayas, Miagao, Iloilo, Philippines; 7 West of Scotland Specialist Virology Centre, Glasgow, United Kingdom; 8 Indian Immunologicals Ltd (IIL), Rakshapuram, Gachibowli Post, Hyderabad Telangana, India; 9 Drug Discovery Unit (DDU), Wellcome Centre for Anti-Infectives Research, School of Life Sciences, University of Dundee, Dundee, United Kingdom; 10 School of Medical Sciences, Griffith University, Gold Coast, Queensland, Australia; Centre International de Recherche en Infectiologie (CIRI), FRANCE

## Abstract

The recent emergence of Severe Acute Respiratory Syndrome Coronavirus 2 (SARS-CoV-2), the underlying cause of Coronavirus Disease 2019 (COVID-19), has led to a worldwide pandemic causing substantial morbidity, mortality, and economic devastation. In response, many laboratories have redirected attention to SARS-CoV-2, meaning there is an urgent need for tools that can be used in laboratories unaccustomed to working with coronaviruses. Here we report a range of tools for SARS-CoV-2 research. First, we describe a facile single plasmid SARS-CoV-2 reverse genetics system that is simple to genetically manipulate and can be used to rescue infectious virus through transient transfection (without in vitro transcription or additional expression plasmids). The rescue system is accompanied by our panel of SARS-CoV-2 antibodies (against nearly every viral protein), SARS-CoV-2 clinical isolates, and SARS-CoV-2 permissive cell lines, which are all openly available to the scientific community. Using these tools, we demonstrate here that the controversial ORF10 protein is expressed in infected cells. Furthermore, we show that the promising repurposed antiviral activity of apilimod is dependent on TMPRSS2 expression. Altogether, our SARS-CoV-2 toolkit, which can be directly accessed via our website at https://mrcppu-covid.bio/, constitutes a resource with considerable potential to advance COVID-19 vaccine design, drug testing, and discovery science.

## Abstract 2

To help meet the ensuing demand for COVID-19 reagents, this article presents an openly available ‘coronavirus toolkit’ (https://mrcppu-covid.bio) and describes the generation and validation of these tools, including a simple SARS-CoV-2 reverse genetics system and a near-comprehensive panel of SARS-CoV-2 antibodies.

## Introduction

Severe Acute Respiratory Syndrome Coronavirus 2 (SARS-CoV-2) is the causative agent of the Coronavirus Disease 2019 (COVID-19) pandemic. SARS-CoV-2 emerged in late 2019 in the western Chinese city of Wuhan, in Hubei Province, and following a rapid and explosive outbreak, spread to over 175 countries in the following months [1]. Despite an unprecedented and globally coordinated response to stop the pandemic, over 92 million cases and 1.9 million deaths (as of January 14, 2021) have so far been attributed to COVID-19 [2]. SARS-CoV-2 is a member of the *Betacoronavirus* genus and is closely related to the SARS-CoV which caused an outbreak of acute viral pneumopathy in 2002 to 2003 [3,4]. Data gathered throughout the 2020 COVID-19 pandemic indicate that SARS-CoV-2 is more transmissible than SARS-CoV [5], reinforcing the need for effective therapeutic and preventative measures, including vaccines, antivirals, and anti-inflammatory drugs to limit viral replication and/or acute lung inflammation. To date, a number of vaccine candidates, including an mRNA-based vaccine [6] and a chimpanzee adenovirus-vectored vaccine (ChAdOx1-nCoV19) [7–9] have undergone Phase III clinical trials and showed effective protection, including in patients over 65 years old. Likewise, monoclonal antibody treatments aimed at neutralising viral particles as a therapeutic intervention for adult COVID-19 patients have recently been granted emergency FDA approval [10–12].

The successful identification and characterisation of these and other SARS-CoV-2 vaccine or drug candidates will require extensive characterisation of SARS-CoV-2 in vivo and in vitro, and this in turn will rely upon validated laboratory resources to facilitate this research. Coronaviruses possess a particularly large RNA genome, where the replicase functions are carried out by numerous nonstructural proteins (nsp), arising from cleavage of 2 large replicase polyproteins (termed ORF1a and ORF1b, respectively) [4]. Consequently, replication and transcription of the genome is complex. Currently, a limited number of tools, including reverse genetics (RG) strategies, have been described for this virus. The current RG systems are often based on end-ligation cloning strategies and are reliant on in vitro transcription, with constructs expressing nucleocapsid protein provided in *trans* [13–15]. Moreover, the existing “full length” yeast artificial chromosome (YAC) systems still require in vitro transcription coupled with nucleocapsid expression in *trans* to rescue infectious virus [14]. Similarly, a recently described bacterial artificial chromosome (BAC)-based infectious clone, though competent both in vitro and in an in vivo hamster model of SARS-CoV-2 infection [16], is devoid of reporter cassettes that, when inserted with fluorescent protein tags, markedly facilitate the visualisation of infected cells and tissues. Here, we describe a variety of tools to facilitate SARS-CoV-2 research. First, we describe a user-friendly RG plasmid system, in which an infectious cDNA (icDNA) clone of SARS-CoV-2, based on the Wuhan strain (Wuhan-Hu-1), was generated using a low copy plasmid backbone (which facilitates rapid and straightforward genetic manipulation). To increase the functionality of this system, we used conventional methods to insert a variety of reporter cassettes (e.g., mCherry, ZsGreen, and Nanoluciferase (NLuc)) into the functional icDNA genome. Because the viral genome is flanked by a eukaryotic promoter and terminator, we show that infectious virus can be rescued by transient transfection of a single plasmid species (with no DNA ligation or in vitro transcription required). Using this system, we rescued infectious virus, at the first attempt, in 3 different countries (which, crucially, had not worked with CoVs prior to 2020).

We further describe the production of a near-comprehensive panel of SARS-CoV-2 antibodies (targeting all but 3 SARS-CoV-2 proteins) alongside a smaller panel of antibodies targeting key SARS-CoV, Middle East Respiratory Syndrome Coronavirus (MERS-CoV), and seasonal human coronavirus (HCoV) OC43 and 229E antigens. We demonstrate the utility of these antibodies in a multitude of functional assays, such as western blotting (WB), immunofluorescence (IF), and co-immunoprecipitation (co-IP) assays. Crucially, these antibodies can reveal the abundance and location of most SARS-CoV-2 proteins at different stages of viral replication, and through this approach, we provided what was, to the best of our knowledge (at the time of first submission), the first evidence supporting protein expression of ORF10 (which has no ortholog in SARS-CoV). In addition, this resource also includes multiple clinical isolates, as well as modified cell lines with increased susceptibility to SARS-CoV-2 infection, which we have used to reveal the TMPRSS2-dependence of the potent anti-SARS-CoV-2 activity of apilimod.

As many labs worldwide pivot to identify drug and vaccine candidates to tackle COVID-19, the resources and technologies described herein aim to accelerate COVID-19 research. Most importantly, this coronavirus toolkit, including SARS-CoV-2 infectious clones, patient isolates, cell lines, and antibodies, has been made openly available to research groups worldwide through our user-friendly webpage (https://mrcppu-covid.bio/) and established biorepositories.

## Results

### Design of SARS-CoV-2-Wuhan-Hu1 clone and rescuing of icDNA-derived SARS-CoV-2

Because coronavirus genomes are substantially longer than those of most RNA viruses, many coronavirus RG systems involve techniques not normally used in RNA virus rescue. Specifically, the in vitro transcription of multiple ligated cDNAs or the propagation of YACs are not necessary for rescuing most RNA viruses. Thus, we sought to construct a simple and accessible single-plasmid rescue system to accelerate COVID-19 research (particularly in laboratories unaccustomed to working with coronaviruses).

We designed our SARS-CoV-2 infectious cDNA clone based on the Wuhan-Hu-1 isolate and the viral cDNA was cloned into pCC1-4K, a derivative of the pCC1BAC (EPICENTRE). Importantly, a substantial fraction of the regulatory sequences was removed to make the pCC1-4K plasmid (which lacks an intact F factor). In the correct strains, the pCC1-4K system is maintained at a single copy per cell. Because coronavirus-derived cDNA sequences can be unstable when propagated in bacteria, the low copy number facilitates the simple genetic manipulation of SARS-CoV-2 icDNA. Importantly, following successful genetic manipulation, the presence of an arabinose-inducible origin in pCC1 means that supplementing bacterial cultures with arabinose can greatly enhance DNA yield, and therefore large quantities of transfection-quality DNA can be prepared using standard reagents.

**Fig 1:**
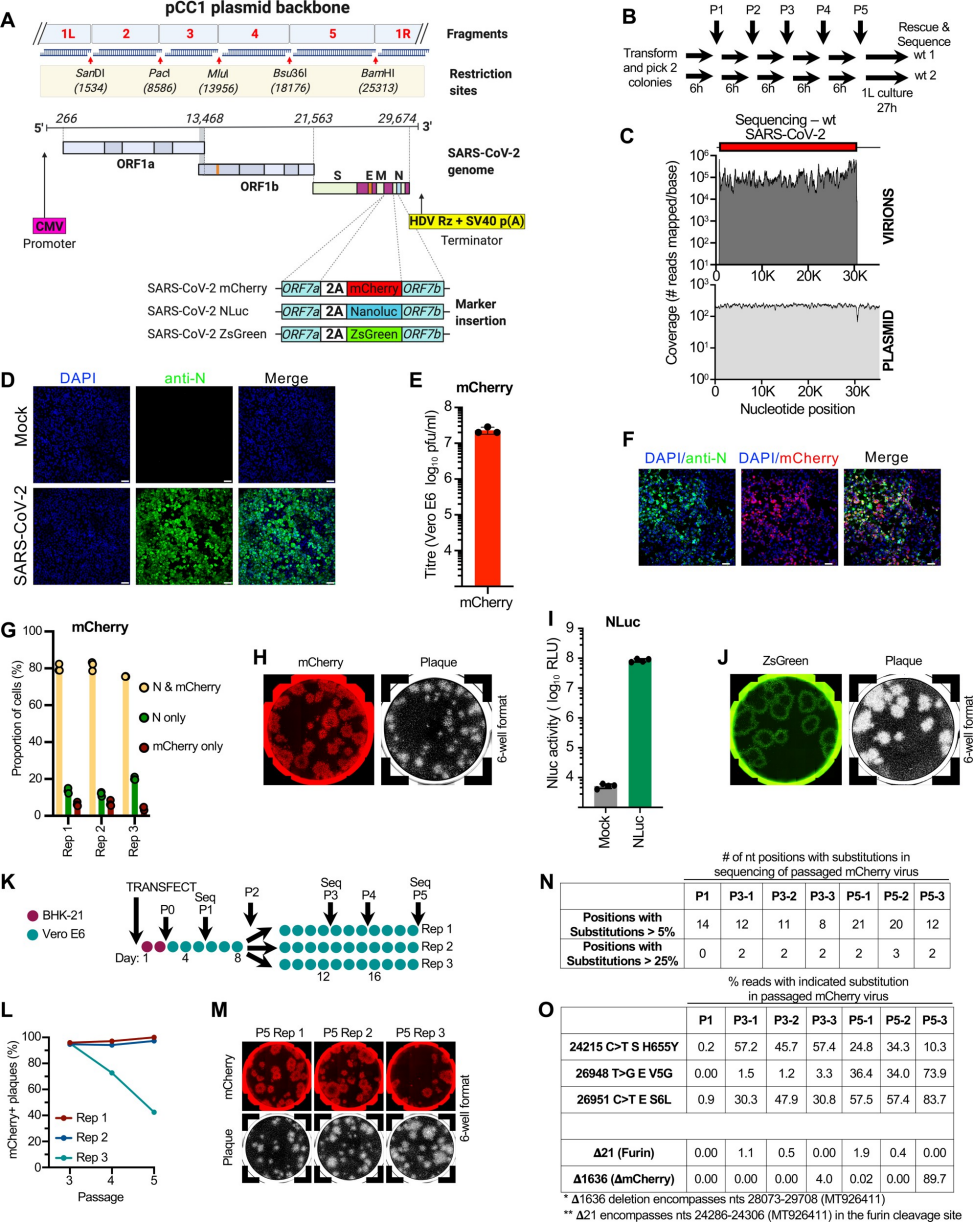
An openly available plasmid launched SARS-CoV-2 RG system. (A) Schematic of the design and construction of a pCC1-4K-SARS-CoV-2-Wuhan-Hu-1 icDNA clone. Synthetic DNA fragments 1, 2, and 3 based on the SARS-CoV-2-Wuhan-Hu1 sequence were cloned into the pCC1 plasmids, and fragments 4 and 5 were cloned into high copy plasmid pUC57Kan by the gene synthesis company (Genscript). Fragments were designed to contain specific restriction cloning sites *San*Dl, *Pac*I, *Mlu*l, *Bsu36*I, and *Bam*HI for cloning purposes. Sequences encoding for mCherry, ZsGreen, and NLuc markers were cloned in-frame to the C-terminus of the ORF7a protein via an FMDV 2A linker. (B) A summary of the passage (P) history of the extensively propagated wt 1 and wt 2 plasmid preps (expanded 6 times on solid agar and 5 times in liquid culture prior to being “grown up” in liquid culture for DNA extraction). (C) Summary plots of the number of reads mapping to the wt rescue plasmid and to the rescued viral genome. (D) Detection of SARS-CoV-2 N antigen in Vero E6 cells infected with RG-rescued SARS-CoV-2-Wuhan-Hu-1. Cells were not infected (Mock) or infected at MOI 1.0 for 48 h and then fixed and stained with mouse monoclonal anti-N antibody and counterstained with DAPI. Cells were imaged using a confocal microscope. Scale bar = 50 μm. (E) Titre of the mCherry virus (plaque forming units on Vero E6 cells) following propagation in Vero E6 cells (P3). (F) Detection of infected Vero E6 cells using the RG-rescued SARS-CoV-2-mCherry. Cells were infected for 48 h at MOI 1.0, then fixed, stained with anti-N antibody, and imaged as in (D). Scale bar = 50 μm. (G) Quantification of mCherry and N expression in Vero E6 cells infected with SARS-CoV-2-mCherry at an MOI of 0.1 for 48 h. Fixed and permeabilised cells were stained for N protein and with Hoechst 33342 (2 μg/ml). Cells were imaged using the Celigo Imaging Cytometer to identify the proportion of infected cells positive for mCherry and/or N protein. (H) Fixed plaque assays were scanned using a Celigo Imaging Cytometer (Nexcelom Bioscience) using the red channel to visualise mCherry. The cells were then subsequently stained with Coomassie staining solution and imaged again. (I) Detection of viral replication in infected Vero E6 cells using the RG-rescued SARS-CoV-2-NLuc. Cells were not infected (Mock) or infected at MOI 1.0 for 24 h, then lysed. NLuc activity was measured in the lysate using a luminometer. (J) Fluorescent plaques of the SARS-CoV-2-ZsGreen virus were visualised using the green channel as in panel H. (K) A schematic of the passage (P) history of the SARS-CoV-2 mCherry virus used to assess reporter stability. Each filled circle represents 1 day of propagation. (L) Plaque assays of passages 3 to 5 were scanned (Celigo) as in (H). The percentage of plaques visible following Coomassie staining that were mCherry-positive in the linear range of the dilution series (total plaque number in the range of 22 to 55 for each replicate) are plotted for each lineage at each passage. (M) Typical images of fluorescent plaques used for the quantification in (L) are shown. (N) A summary of the variation generated during the passage of the mCherry virus summarised in panel K. The variation is detailed in S1 Table. (O) The percentage of the viral swarm displaying all variants that exceed 25% of the swarm at any time point is shown. The low-level deletion of the furin cleavage site is also highlighted for interest. The data underlying Fig 1E, 1G, 1I and 1L may be found in S1 Data. FMDV, foot-and-mouth disease virus; icDNA, infectious cDNA; MOI, multiplicity of infection; NLuc, Nanoluciferase; RG, reverse genetics; SARS-CoV-2, Severe Acute Respiratory Syndrome Coronavirus 2; wt, wild-type.

We assembled the SARS-CoV-2 icDNA genome from 5 synthetic fragments, each flanked by unique restriction sites *San*DI (1524), *Pac*I (8586), *Mlu*I (13956), *Bsu*36I (18176), and *Bam*HI (25313) (**Fig 1A**). A human cytomegalovirus (CMV) promoter was inserted at the position corresponding to the 5′ end of the genome, and a dual hepatitis delta virus (HDV) ribozyme and Simian Virus 40 terminator (SV40) were added downstream of the poly-A tail at the 3′ end of the virus genome. These elements ensure efficient transcription and homogenous 3′ end processing during the rescue of infectious virus. To enable direct visualisation and quantification of replicating virus in cells, we also generated variants containing sequences encoding for fluorescent (mCherry and ZsGreen) and bioluminescent (Nanoluciferase, or NLuc) markers inserted into the plasmid backbone. To avoid deletion of viral sequences, the markers were cloned in-frame after the C-terminus of the ORF7a protein using a foot-and-mouth disease virus (FMDV) 2A “ribosomal skipping” linker (2A) to liberate the nascent reporter protein from the ORF7a protein (**Fig 1A**).

Because plasmid clones harbouring the full-length SARS-CoV-2 genome could be unstable, we transformed the wild-type (wt) rescue plasmid, picked 2 colonies, and passaged each lineage through a series of 5 liquid cultures (summarised in **Fig 1B**). Bacterial cells were plated in between each passage, and a single colony was used to initiate each subsequent liquid passage. To validate plasmid integrity, a single colony derived from each lineage of the fifth liquid passage was chosen at random and “grown up” (1 litre culture induced with arabinose), and the culture used to extract plasmid DNA. The plasmids were then sequenced with an average coverage of 649 reads (wt 1) and 829 reads (wt 2) (S1 Fig). Using a conservative estimate of 1 duplication per hour of culture, the 2 sequenced colonies underwent over 100 divisions and both colonies (out of over 1 × 10^30^ progeny) yielded DNA with the correct consensus sequence. There were no subconsensus substitutions detected at a frequency greater than 5% and in this approximately 35 kb construct, only 2 substitutions were detected in each lineage at a frequency >1%. Thus, this plasmid system is stable for functional use. To rescue icDNA-derived infectious viruses, we transfected BHK-21 cells with icDNA clones of SARS-CoV-2, alongside derivatives harbouring mCherry, ZsGreen, and NLuc markers. The following day, culture supernatant was used to inoculate subconfluent monolayers of Vero E6 cells. Following 4 days of culture, the virus-containing supernatant was harvested and the viral RNA was extracted and sequenced (available at GenBank BioProject PRJNA658321). This confirmed that “full-length” viruses had been rescued and that no nucleotide substitutions had accumulated during the rescue and amplification steps. As expected, the frequency of subconsensus substitutions was higher in the rescued viruses with 4 to 15 substitutions detected at a frequency >5%. Crucially, the sequencing reads of the rescued viruses overwhelmingly mapped to the viral genome (and not the flanking plasmid) indicating that we sequenced rescued viral RNA and not contaminating plasmid DNA (**Fig 1C** and S1 Fig).

The resulting RG-rescued SARS-CoV-2 viruses replicated efficiently and could readily be detected by IF microscopy using a commercial anti-SARS-CoV-2 nucleocapsid (N) antibody (**Fig 1D**). Moreover, the rescued viruses induced cytopathic effect (CPE) and can be used in conventional plaque assays (**Fig 1E**). When reporter expression and antigen expression were compared, approximately 80% of infected Vero E6 cells expressed the reporter and N (**Fig 1F and 1G**). The apparent 10% to 20% of infected cells that expressed N but not mCherry (**Fig 1G**) likely reflected asynchrony between N and mCherry expression as >95% of plaques from the same passage were mCherry positive (**Fig 1H**). The NLuc and ZsGreen reporter systems also facilitated the detection and quantification of recombinant SARS-CoV-2 replication in infected cells. SARS-CoV-2-NLuc provided a robust readout of over 4 orders of magnitude of bioluminescence intensity (**Fig 1I**), and SARS-CoV-2-ZsGreen plaques were all ZsGreen positive at early passages (**Fig 1J**).

Reporter viruses are usually less replicatively fit than their unmodified counterparts, which can lead to reporter-negative variants emerging during extended propagation. To assess the stability of the *ORF7a*-2A-Reporter cassette, we passaged the SARS-CoV-2-mCherry virus 5 times in Vero E6 cells (**Fig 1K**). After 5 passages (P5), >95% of plaques were mCherry positive in 2 out of 3 replicates (**Fig 1L** and **1M**). In one of the replicates, however, an mCherry-negative population emerged by P4 and appeared to outcompete the parental mCherry virus (**Fig 1L** and **1M**). To better characterise this process, we sequenced P2, P3, and P5 viral swarms (**Fig 1N** and **1O**). By P3, a deletion mutant was detected at low frequency (4%) that had lost 1,636 nucleotides (**Δ**1,636) encompassing the last 2 codons of *ORF6* and all of *ORF7a*, all of *mCherry*, all of *ORF7b*, and the majority of *ORF8* (the last 2 codons remain). By P5, this variant accounted for the majority of the viral swarm (approximately 60% to 90%). Thus, although the majority of the replicates of the reporter viruses appear stable over many passages, deletion variants can rapidly overrun the culture once they arise.

### Generation of coronavirus antibodies

**Fig 2:**
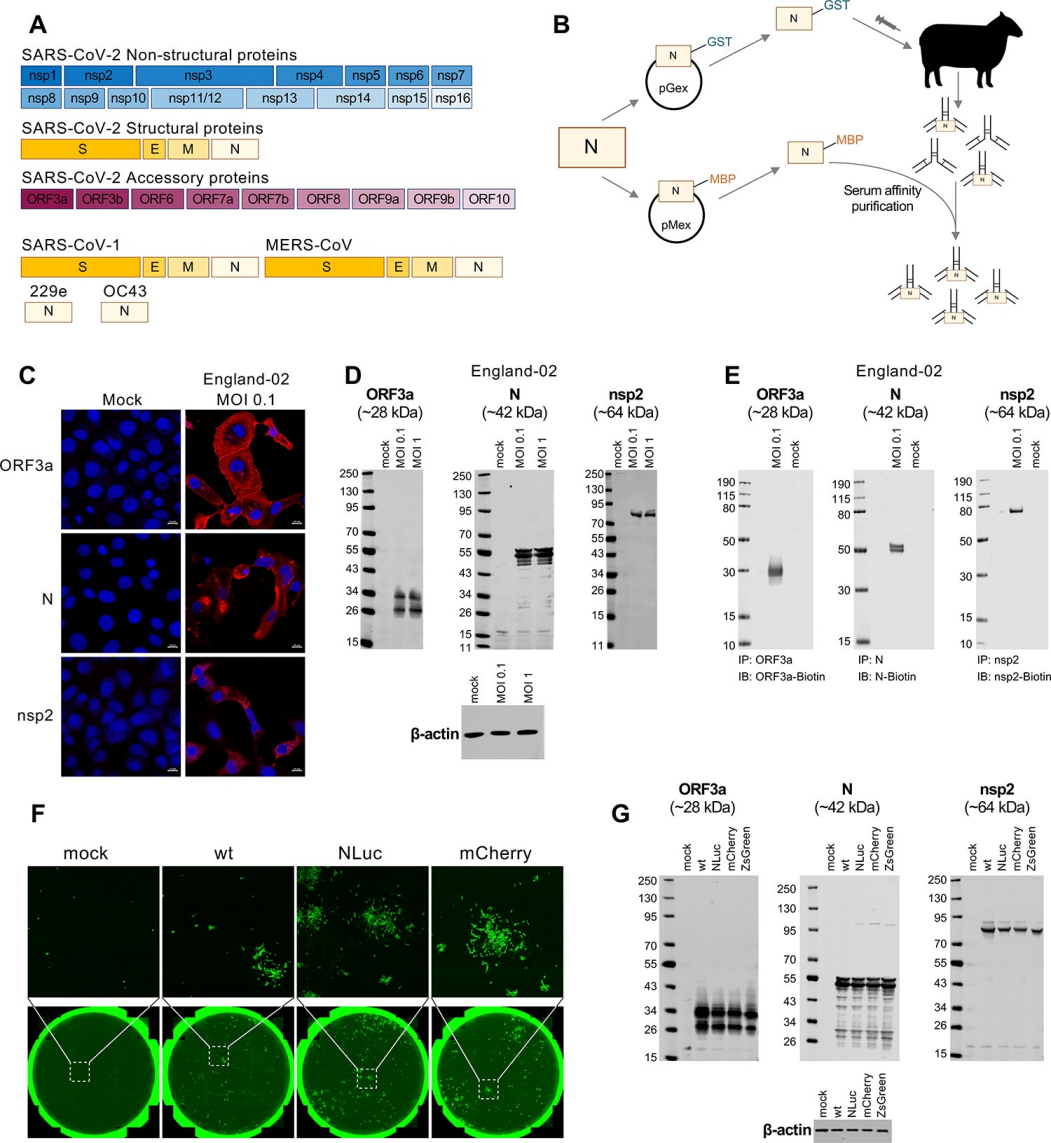
Production and validation of a near-comprehensive panel of openly available SARS-CoV-2 and coronavirus antibodies. (A) Schematic representation of the SARS-CoV-2 and other coronavirus-processed proteins for which antibody synthesis and validation is described in this study. All antibodies (and corresponding proteins and cDNAs) listed are available upon request at https://mrcppu-covid.bio/. Accession numbers for the sequences utilised can be found in S2 Table and at https://mrcppu-covid.bio/. (B) A schematic illustrating the method of production for the antibodies shown in (A), utilising N (nucleocapsid) as an example. Each cDNA encoding a coronavirus protein was cloned into both pGex (carrying a GST tag) and pMex (MBP tag) plasmids, in order to yield corresponding GST- and MBP-tagged viral proteins. Sheep were subsequently immunised by utilising the GST-tagged proteins as antigens. At day 7 postimmunisation, serum was harvested and antibodies were affinity purified using the corresponding MBP-tagged protein. Additional inoculations (up to 5 in total) occurred 28 days apart. Exceptions to this method occurred for SARS-CoV-2 ORF7a, S, and S-RBD antibodies, where the MBP-tagged proteins were used as antigens, and after harvest, serum was again purified against the MBP-tagged protein, followed by depletion of the antibody against MBP. The SARS-CoV-2 S antibody was purified using protein G Sepharose chromatography. (C) IF validation for three of the antibodies (against nonstructural nsp2, structural N, and accessory ORF3a proteins) shown in (A) was conducted in Vero E6 cells that were uninfected (mock) or infected with the SARS-CoV-2 England-02 virus at an MOI of 0.1 for 48 h prior to fixation, permeabilization, and staining. IF validation for the remaining antibodies in (A) is shown in S2 Fig. (D) WB validation for the 3 antibodies shown in (C) was conducted using Vero E6 cells that were uninfected (mock) or infected with England-02 virus at an MOI of 0.1 or 1 (as indicated) for 72 h followed by WB analysis of whole cell lysates. WB validation for the remaining antibodies in (A) is shown in S3 and S4 Figs. (E) IP validation for the 3 antibodies shown in (C) was conducted using Vero E6 cells that were uninfected (mock) or infected with England-02 virus at an MOI of 0.1 for 3 days. IP validation for the remaining antibodies in (A) is shown in S4 and S5 Figs. (F) Immunostaining of cells either uninfected (mock) or infected with the RG-rescued SARS-CoV-2 and its mCherry and NLuc derivatives. Cells were stained using the N antibody utilised in (C–E) and imaged using a Celigo imaging cytometer. (G) WB validation of Vero E6 cells infected for 48 h with the RG viruses shown from Fig 1A for 48 h, using the 3 antibodies shown in (C–E). GST, glutathione S-transferase; IB, immunoblotting; IF, immunofluorescence; IP, immunoprecipitation; MBP, maltose-binding protein; MERS-CoV, Middle East Respiratory Syndrome Coronavirus; MOI, multiplicity of infection; NLuc, Nanoluciferase; RG, reverse genetics; SARS-CoV-2, Severe Acute Respiratory Syndrome Coronavirus 2; WB, western blotting; wt, wild-type.

There is an urgent need to develop antibodies specifically targeting SARS-CoV-2 proteins to accelerate COVID-19 research. We therefore generated a near-comprehensive panel of sheep polyclonal antibodies against the structural, nonstructural, and accessory proteins of SARS-CoV-2. In addition, we raised antibodies to the structural proteins of SARS-CoV and MERS-CoV (S, E, M, and N proteins), as well as the N proteins of HCoV 229E and HCoV OC43 (**Fig 2A**). To maximise the usefulness of these tools to the global scientific community, we sought to experimentally validate these antibodies through established assays (although at the time of submission, SARS-CoV-2 nsp4 and nsp6, and SARS-CoV and MERS-CoV S and M antibodies were not yet available for validation).

The generation of the antibodies is illustrated in **Fig 2B**. Briefly, individual glutathione S-transferase (GST)-tagged SARS-CoV-2 proteins were used to inoculate sheep, and immunoglobulin G (IgG) was subsequently isolated and affinity purified using maltose-binding protein (MBP)-tagged variants of each viral protein. To validate our panel of antibodies, we initially examined their utility in indirect IF confocal microscopy. Vero E6 cells were mock infected or infected with SARS-CoV-2 England-02 (from Public Health England) at multiplicity of infection (MOI) 0.1 for 48 h, and then fixed and stained with each antibody. Antigen staining was highly specific to SARS-CoV-2-infected cells, exemplified by the staining of ORF3a, nsp2, and N proteins (**Fig 2C**). Antibodies raised against the other SARS-CoV-2 proteins were also highly specific, and more importantly, revealed the distinct staining and spatial distribution profiles of most SARS-CoV-2 proteins (S2 Fig).

In a similar fashion, we validated our panel of antibodies targeting SARS-CoV-2 proteins using WB. Vero E6 cells were infected with the England-02 isolate of SARS-CoV-2 at MOI 0.1 or 1 (as indicated) for 72 h (to ensure that most of the culture was infected using both multiplicities). Importantly, we were able to detect all available SARS-CoV-2 proteins using our antibodies, again exemplified by ORF3a, N, and nsp2 (**Fig 2D)** with the data for all remaining antibodies shown in S3 Fig. Interestingly, previous work has proposed that the ORF10 protein is unlikely to be expressed and may be a misannotation [17]. Remarkably, ORF10 was specifically detected in infected cells by WB and IF using our antibodies (S2 and S3 Figs).

Furthermore, in the small number of instances examined, substantial cross-reactivity of the antibodies was observed. For example, the antibodies raised against SARS-CoV N, MERS-CoV N, and HCoV OC43 N efficiently recognised SARS-CoV-2 N, whereas cross-reactivity was not observed for antibodies raised against N from the alphacoronavirus HCoV 229E (S4 Fig).

In addition to IF and WB, we examined the utility of our panel of antibodies in immunoprecipitation (IP) assays. Here, Vero E6 cells were infected with England-02 for 3 days at MOI 0.1. Interestingly, the antibodies generally appeared more sensitive in IP assays than WB, and anti-HCoV 229E N cross-reactivity with SARS-CoV-2 N that was not observed by WB was evident by IP (S4 Fig). Although cross-reactivity was present for SARS-CoV E, no cross-reactivity was observed by IP or WB using anti-MERS-CoV E and SARS-CoV-2 antigens (S4 Fig). Importantly, the antibodies resulted in efficient and specific IP of all but one (envelope [E]) of the SARS-CoV-2 proteins we raised antibodies to (**Fig 2E** and S5 Fig).

Next, we validated our SARS-CoV-2 N antibody in cells infected with the icDNA-derived viruses. Vero E6 cells were infected with the RG-rescued SARS-CoV-2-Wuhan-Hu-1 (wt), SARS-CoV-2-NLuc, or SARS-CoV-2-mCherry for 48 h, and N protein was detected by IF (**Fig 2F**). Similarly, ORF3a, nsp2, and N proteins were detected in Vero E6 cell lysates after infection with all the RG viruses from Fig 1A, confirming the utility of our antibody panel (**Fig 2G**). As nearly all antibodies are compatible with IF, WB, and IP, they represent an invaluable resource to researchers investigating SARS-CoV-2 and COVID-19.

**Fig 3:**
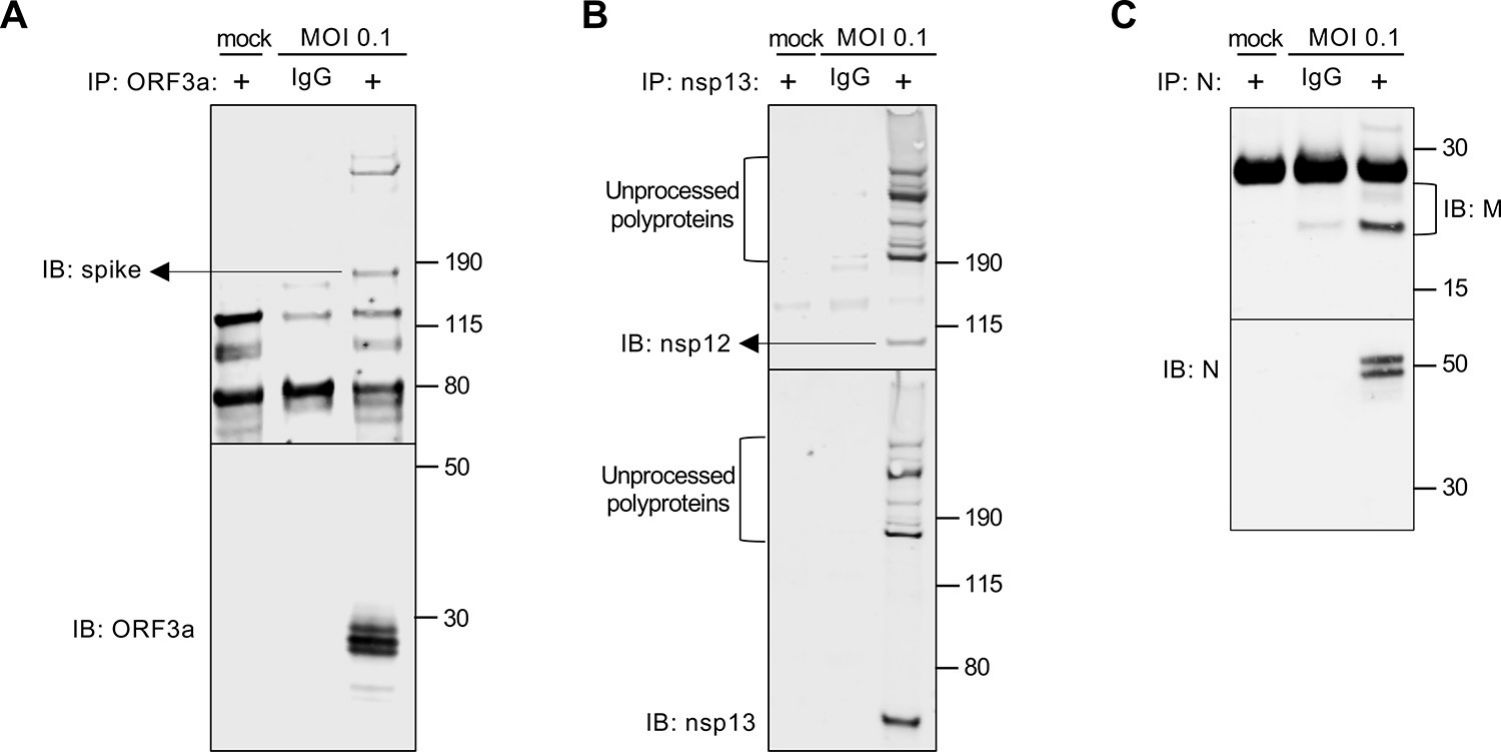
Usage of our toolkit antibodies to demonstrate or confirm SARS-CoV-2 protein interactions. (A) A co-IP was performed using lysates from Vero E6 cells infected with England-02 at MOI 0.1 for 3 days. Using the specific anti-ORF3a antibody described herein, SARS-CoV-2 ORF3a was immunoprecipitated (alongside a preimmune IgG control), and the immune complexes were western blotted for the presence of SARS-CoV-2 spike (S) and ORF3a. (B) As in (A), SARS-CoV-2 nsp13 (or IgG control) was immunoprecipitated and the immune complexes were probed for nsp13 and nsp11/12 by WB. (C) As in (A, B), SARS-CoV-2 N (or IgG control) was immunoprecipitated and the immune complexes were probed for matrix (M) and N by WB. co-IP, co-immunoprecipitation; IB, immunoblotting; IgG, immunoglobulin G; MOI, multiplicity of infection; SARS-CoV-2, Severe Acute Respiratory Syndrome Coronavirus 2; WB, western blotting.

We next investigated whether our antibody panels could be a useful tool in the study of protein–protein interactions, for which co-IP is a common technique. We performed co-IPs to look at interactions between different viral proteins based on studies performed with other CoVs [18–21]. Using lysates from infected Vero E6 cells (England-02, MOI 0.1), we first immunoprecipitated ORF3a protein using our ORF3a antibody and probed for the presence of spike (S) protein and ORF3a (**Fig 3A**) [18–21]. This revealed a predicted interaction between SARS-CoV-2 S and the accessory protein ORF3a, that had been observed in SARS-CoV. Likewise, we performed co-IPs and confirmed interactions between the SARS-CoV-2 nsp12 and nsp13 proteins (**Fig 3B**) [22]. In addition, we revealed a predicted interaction between SARS-CoV-2 N and M proteins (**Fig 3C**). Altogether, our coronavirus toolkit’s near-comprehensive SARS-CoV-2 antibody panel will greatly facilitate the study of coronavirus protein–protein interactions.

### Generation of SARS-CoV-2 permissive cell lines

**Fig 4:**
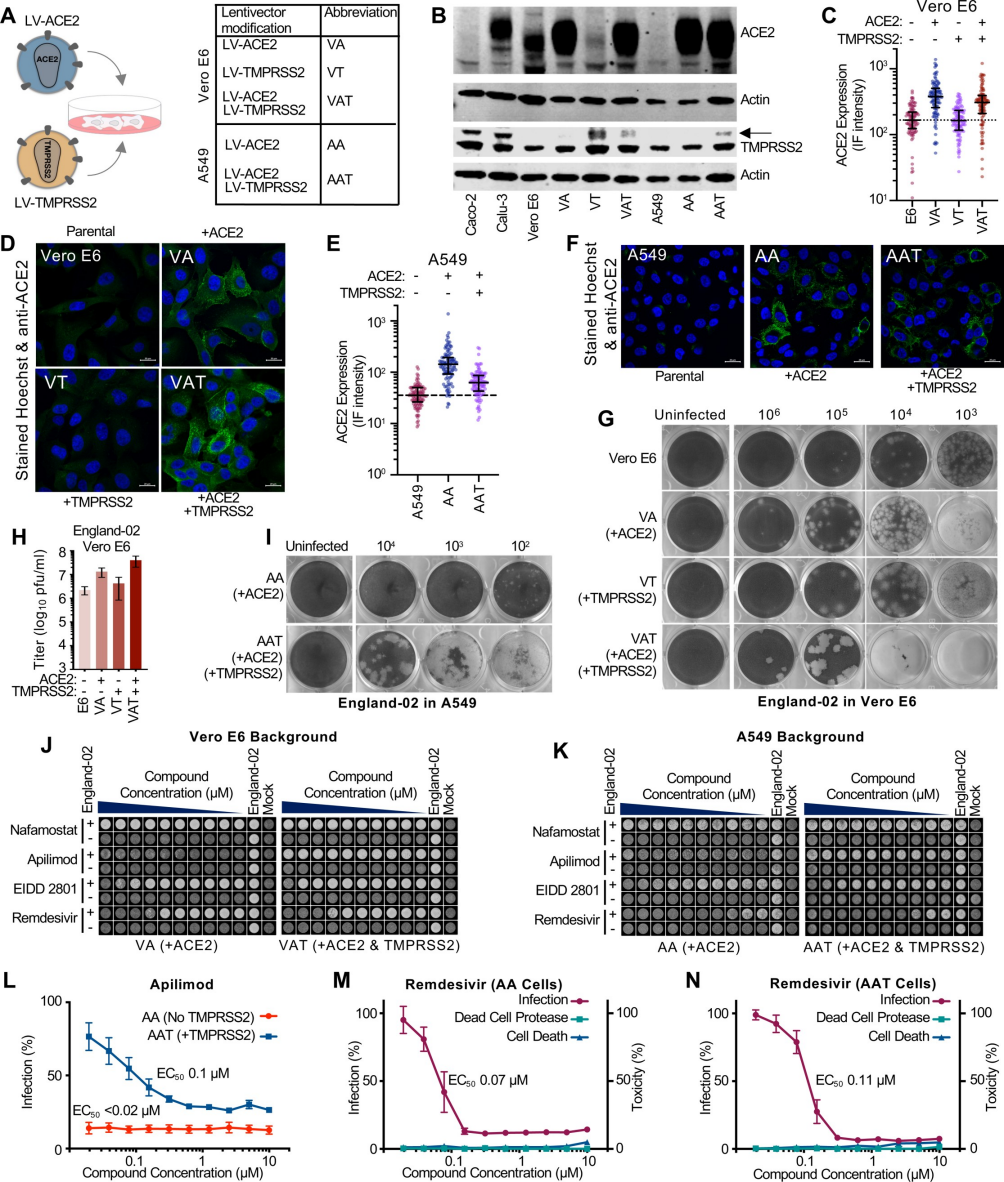
Conventional cell lines modified to express ACE2 and TMPRSS2 lentiviruses have utility in SARS-CoV-2 phenotypic assays. (A) A schematic illustrating the LV-ACE2 and LV-TMPRSS2 lentiviruses that have been used to transduce multiple cell lines. The corresponding abbreviations of the modified lines are also shown. (B) The abundance of ACE2 and TMPRSS2 in Vero E6 cells, A549 cells and derivatives modified to express exogenous ACE2 and/or TMPRSS2 was assessed by WB. Permissive Calu-3 and Caco-2 samples were included for reference. Two actin blots are presented as different samples of equivalent cells were used to stain for ACE2 and TMPRSS2. (C) ACE2 intensity values from confocal microscopy images of ACE2 and Hoechst-stained Vero E6 cells and VA, VT, and VAT derivatives were measured using Cell Profiler (cellprofiler.org). (D) Randomly selected confocal microscopy images used for the quantification in (C) are shown. (E) As in panel C, using stained A549 cells, as well as AA and AAT cell derivatives. (F) Randomly selected confocal microscopy images used for the quantification in (E) are shown. (G) The plaque phenotype produced by the same preparation of SARS-CoV-2 England-02 on Vero E6 cells and VA, VT, and VAT derivatives. (H) As in (G), indicating the titre (in pfu/ml) of the same preparation of SARS-CoV-2 England-02 in cells with or without exogenous ACE2 and/or TMPRSS2. (I) As in panel G, AA and AAT derivatives of A549 cells were infected with SARS-CoV-2 England-02 to observe the plaque phenotype. (J) An example of a phenotypic well clearance/monolayer integrity assay used to assess the anti-SARS-CoV-2 activity of various compounds (nafamostat, apilimod, EIDD_2801, or remdesivir). The cells were treated with 2-fold serially diluted compound (10 μM to 20 nM) before being mock infected or infected with SARS-CoV-2. At 72 h postinfection, the monolayers were fixed and Coomassie-stained before scanning using a Celigo imaging cytometer. (K) As in panel J, well clearance assays in AA and AAT cells using SARS-CoV-2 England-02 are shown. (L) Quantification of the anti-SARS-CoV-2 activity of apilimod in AA and AAT cells. The mean and standard error of 4 replicates are plotted. (M) Dose response curve of remdesivir using the well-clearance assay in AA cells (as in panel K) multiplexed with a dead cell protease toxicity assay. The mean and standard error of 4 replicates are plotted. (N) as in panel M using AAT cells. The data underlying Fig 4C, 4E, 4H, 4L, 4M and 4N may be found in S1 Data. AA, A549-ACE2; AAT, A549-ACE2-TMPRSS2; ACE2, angiotensin-converting enzyme 2; SARS-CoV-2, Severe Acute Respiratory Syndrome Coronavirus 2; VA, Vero E6-ACE2; VAT, Vero E6-ACE2-TMPRSS2; VT, Vero E6-TMPRSS2; WB, western blotting.

SARS-CoV-2 has been shown to infect a variety of cell types, in particular nasal, epithelial, and lung (bronchoalveolar, bronchial, and epithelial) cells [13]. In addition, multiple reports indicate that gut epithelial and brain cells may also be natural targets of infection [23,24]. In humans, angiotensin-converting enzyme 2 (ACE2) is the main receptor and the TMPRSS serine proteases cleave the viral S protein, priming it for infection. Importantly, blockade/inhibition of these proteins has been shown to inhibit viral infection [25]. Because many cell lines derived from human lung are not susceptible to SARS-CoV-2 infection, such as A549 cells, we sought to engineer susceptibility in cell lines that can easily be adapted for many assays (including medium- to high-throughput formats). We transduced Vero E6 and A549 epithelial cell lines using LV lentiviral vectors to generate ACE2 and TMPRSS2-expressing cell lines, as well as double-expressing, ACE2-TMPRSS2 lines (**Fig 4A**). We visualised ACE2 and TMPRSS2 expression by WB (**Fig 4B**) and also assessed ACE2 expression using IF (**Fig 4C–4F**). The modified cell lines expressed ACE2 at higher levels than Calu-3 and Vero E6 cells (**Fig 4B**) and exogenous TMPRSS2 expression reduced ACE2 expression as has been reported previously [26]. Notably, in both cell backgrounds, exogenous ACE2 increased the susceptibility to infection, increasing the clarity of plaques in Vero E6 cells and converting A549s from nonsusceptible to permissive target cells (**Fig 4G–4I**). The exogenous expression of TMPRSS2 alone, in Vero E6 cells, increased the size of the plaques. Interestingly, Vero E6-ACE2-TMPRSS2 (VAT cells) yielded larger and clearer plaques (**Fig 4G**), which were more susceptible to infection as evidenced by the >10-fold increase in pfu/ml relative to unmodified cells (**Fig 4H**). Similar results were observed in AAT (A549-ACE2-TMPRSS2) cells, where exogenous TMPRSS2 expression greatly increased plaque size and clarity, and modestly increased the number of plaques (**Fig 4I**).

Crucially, these modified cell lines are suitable for cytopathic effect (CPE)-based assays. CPE assays can be useful tools in compound screening and compound characterisation, and these cell lines provide a clear output for medium-throughput screening. For illustration, using VA (Vero E6-ACE2) cells, we examined potential inhibitory compounds using conditions where a fixed dose of SARS-CoV-2 would cause substantial CPE, resulting in the cell monolayer being “cleared” in the absence of any inhibitory compound. Virus-induced CPE was effectively abrogated following treatment with apilimod, a phosphatidylinositol-3-phosphate 5-kinase (PIKfyve) inhibitor (**Fig 4J**). In contrast, ribonucleoside analogues remdesivir and EIDD-2801 were less effective, but showed dose-dependent inhibition, while nafamostat, a serine protease inhibitor, displayed no inhibitory effect against SARS-CoV-2. Importantly, the inhibitory effect of apilimod was lost in the presence of TMPRSS2 (VAT cells), whereas the inhibitory effects displayed by remdesivir and EIDD-2801 were unaffected (**Fig 4J**), suggesting that apilimod’s mode of action may be ineffective when TMPRSS2-dependent infection occurs. These observations were recapitulated in AA (A549-ACE2) and AAT cells (**Fig 4K**). The CPE-based assays are also quantitative, allowing dose response curves to be generated and EC_50_ values to be calculated. For example, dose response curves of apilimod inhibition of SARS-CoV-2 in AA and AAT cells are shown in **Fig 4L**. Moreover, the CPE assays can be multiplexed with other assays to monitor compound toxicity in parallel (**Fig 4M and 4N**), and the utility of the AA and AAT cells is indicated in S6 Fig, where these cells were used to consider the ability of a panel of compounds to inhibit SARS-CoV-2.

### Establishment and analysis of clinical isolates

**Fig 5:**
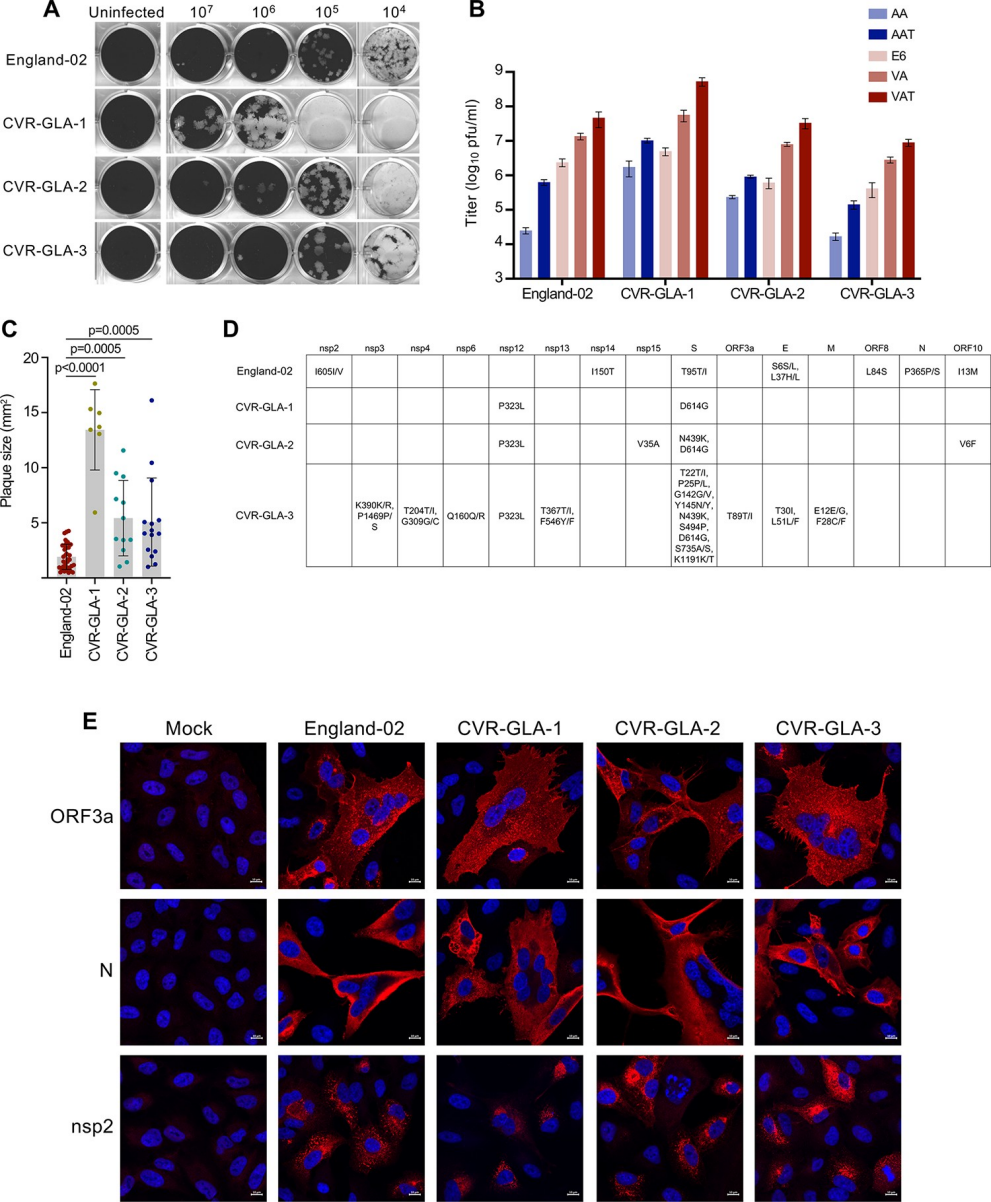
Toolkit of SARS-CoV-2 clinical isolates. (A) A comparison of plaque phenotypes in VAT cells from the 3 SARS-CoV-2 viruses isolated in this study and the England-02 SARS-CoV-2 virus. (B) A comparison of viral titers (in pfu/ml) of the 3 SARS-CoV-2 viruses isolated in this study and the England-02 SARS-CoV-2 virus in AA, AAT, Vero E6 (E6), VA, and VAT cells. All cells with exogenous ACE2 or TMPRSS2 were transduced with the lentiviruses described in Fig 4. (C) Quantification of plaque area (in mm^2^) was calculated in ImageJ (plaques not overlapping other plaques or the side of the well) from 4 replicates of the data presented in (A) and (B). Significance was determined using a Mann–Whitney *U* test. (D) A table showing the amino acid substitutions present in England-02 and 3 SARS-CoV-2 viruses isolated from clinical samples in this study, relative to Wuhan-Hu-1 (NC_045512). CVR-GLA-1 (MT882022) was isolated from sputum (CVR837 [EPI_ISL_461705]), CVR-GLA-2 (MT906650) was isolated from sputum (CVR2224 [EPI_ISL_448167], and CVR-GLA-3 (MT906649) was isolated from bronchoalveolar lavage (CVR3899_BAL [EPI_ISL_490695]). (E) IF of the 3 SARS-CoV-2 viruses isolated in this study and the England-02 SARS-CoV-2 virus in AAT cells (at MOI 0.01 for 48 h), using ORF3a, N, and nsp2 antibodies, as in Fig 2. The data underlying Fig 5B and 5C may be found in S1 Data. AA, A549-ACE2; AAT, A549-ACE2-TMPRSS2; ACE2, angiotensin-converting enzyme 2; IF, immunofluorescence; MOI, multiplicity of infection; SARS-CoV-2, Severe Acute Respiratory Syndrome Coronavirus 2; VA, Vero E6-ACE2; VAT, Vero E6-ACE2-TMPRSS2.

The modified cell lines forming part of this resource are also useful for isolating and characterising SARS-CoV-2 from clinical samples [27]. We have isolated 3 SARS-CoV-2 viruses, CVR-GLA-1, CVR-GLA-2, and CVR-GLA-3, from samples collected from patients hospitalised in Scotland (2 from sputum samples, one from a bronchoalveolar lavage (BAL) sample). Using our modified cell lines to characterise the plaque phenotypes of these isolates, we found that the CVR-GLA virus isolates all caused larger plaques than England-02 (**Fig 5A and 5C**), consistent with the enhanced particle infectivity proposed for the D614G substitution [28]. In addition, while titres across isolates were generally similar to those of England-02, they were increased in the presence of exogenous ACE2 and further increased by the presence of exogenous ACE2 and TMPRSS2, in line with our earlier observations (**Fig 5B**).

Clinical isolates can be particularly useful given the interest in phenotypic variation as the pandemic unfolds, and for this reason, these viruses were sequenced to identify amino acid substitutions relative to Wuhan-1 and England-02 (**Fig 5D**). For example, variants such as D614G in spike (S), which occurs in all of our isolates, have been the subject of intense investigation [28]. Moreover, CVR-GLA-2 exhibits the N439K substitution in S that has also been linked to immune escape from neutralising antibodies [29].

We used our antibody panel to validate the 3 CVR-GLA isolates by IF. AAT cells were uninfected or infected at MOI 0.01 for 48 h with England-02, CVR-GLA-1, CVR-GLA-2, or CVR-GLA-3. The cells were then probed with antibodies against ORF3a, nsp2, and N. As observed in Fig 2C, each of these antibodies displayed strong specificity against their target proteins expressed by all 3 viruses (**Fig 5E**), further validating the reagents generated in this study.

**Fig 6:**
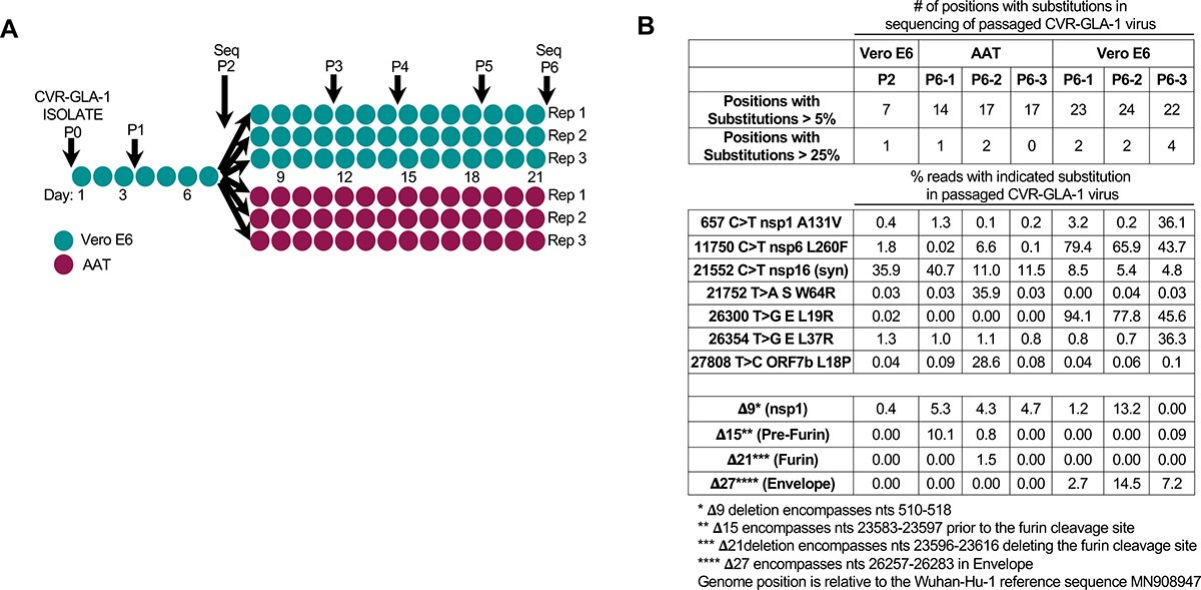
Sequence changes observed in SARS-CoV-2 CVR-GLA-1 following in vitro propagation. (A) A schematic of the passage history of the SARS-CoV-2 CVR-GLA-1 virus in Vero E6 and AAT cells. Each filled circle represents 1 day of propagation. (B) A summary of the variation generated during the passage of the CVR-GLA-1 virus indicated in panel A. The variation is detailed in S1 Table. The percentage of the viral swarm displaying all variants that exceed 5% or 25% of the swarm at any time point is shown. The percentage occurrence of several deletion mutants is highlighted for interest. AAT, A549-ACE2-TMPRSS2; SARS-CoV-2, Severe Acute Respiratory Syndrome Coronavirus 2.

Because most viruses become tissue culture-adapted when they are extensively passaged in vitro, we sought to evaluate whether the human AAT cells might be an alternative substrate to African green monkey Vero E6 cells for amplifying SARS-CoV-2. We passaged the CVR-GLA-1 isolate on Vero E6 cells and AAT cells (**Fig 6A**) and sequenced the P2 and P6 swarms (**Fig 6B**). Strikingly, there was little overlap between the variants observed during passage of the mCherry (**Fig 1N** and **1O** and S1 Table) and GLA-1 (**Fig 6B** and S1 Table) viruses in Vero E6 cells, making it difficult to draw generalisable conclusions. We specifically examined the furin cleavage site in our sequencing data as variation in this region has received a lot of attention. The multibasic S1/S2 furin cleavage site in SARS-CoV-2 S is essential for the infection of human lung cells [30], and multiple studies have reported that this site can be rapidly lost during passage in Vero E6 cells [31,32], likely driven by the enhanced growth of furin deletion mutants in Vero E6 cells [33]. In the 6 lineages of SARS-CoV-2-mCherry and CVR-GLA-1 that were propagated in Vero E6 cells, only 1 lineage out of 6 exhibited a deletion of the furin site at >0.5% abundance. Notably, in lineages that did contain a furin cleavage site deletion (2/6), the deletion variant did not overrun the culture (and even decreased slightly in frequency in 1 lineage), suggesting that these mutants did not have a substantial growth advantage in Vero E6 cells. Surprisingly, while CVR-GLA-1 passaged in AAT cells only lost the furin cleavage site in 1 of 3 replicates (at 1.46%), 1 replicate did contain a deletion mutant near this site at a frequency of approximately 10%. Thus, the AAT cells appear worse than the Vero E6 cells at preserving the integrity of the SARS-CoV-2 furin cleavage site during passage.

These data highlight the heterogeneous nature of SARS-CoV-2 propagation, where virus strain, cell culture conditions, and passaging protocol all likely influence lab-to-lab variation of virus stocks. Moreover, these data underscore the importance of facile, robust, high fidelity rescue systems (such as the plasmid system described herein) for generating uniform SARS-CoV-2 virus stocks that avoid the variation introduced when viral stocks are extensively passaged.

## Discussion

The COVID-19 pandemic has spurred unprecedented global efforts aimed at identifying vaccine candidates, antiviral compounds, or anti-inflammatory strategies to prevent SARS-CoV-2 infection or limit pathology. One of the most important factors driving this fast-paced field is the large-scale scientific collaboration and sharing of data emanating from outbreak sites since the start of the pandemic [14,15,34,35]. Further experimental work requires access to clinical isolates and systems that allow genetic manipulation of SARS-CoV-2. In this report, we describe the development of a research toolkit for studying SARS-CoV-2. First, we showed the generation of a plasmid-launched RG system for SARS-CoV-2 assembled using publicly available sequences of the original SARS-CoV-2 Wuhan-Hu-1 virus isolate. By using an inducible single-copy plasmid backbone, the cDNA of the full-length genome can be stably amplified. Moreover, this single plasmid approach dispenses of the need for approximately 30 kb of error-prone in vitro transcription and assembly of multiple DNA fragments, and it does not require N expression in *trans*. The result is a highly versatile system that simply requires transfection of DNA into cells, after which replication-competent SARS-CoV-2 can be harvested from cell culture supernatants. Notably, during this study, infectious virus was rescued at the first attempt in multiple laboratories (in 3 different countries) that had not previously rescued infectious CoVs. Furthermore, this SARS-CoV-2 construct is readily amenable to genetic manipulation (to study variants) and insertion of reporters, such as fluorescent or bioluminescent proteins, that can be used in a variety of in vitro and in vivo assays and enable direct detection and quantification of in-cell viral replication kinetics. Importantly, the ease of manipulation of the constructs described herein is best exemplified by the fact that the reporter viruses described herein were constructed in multiple laboratories that had no prior experience of generating CoV reporter systems.

As a further part of our SARS-CoV-2 coronavirus toolkit, we describe a near-comprehensive panel of antibodies targeted against nearly all SARS-CoV-2 proteins and key structural proteins of SARS-CoV and MERS-CoV. These antibodies demonstrated high specificity, are compatible with the “go-to” virological techniques optimised for the detection and quantification of viral infection (e.g., IF and WB), and are also very useful in probing viral protein–protein interactions. Indeed, these antibodies have already been used in high-profile COVID-19 publications [36,37]. As an additional example of the utility of these antibodies, we confirmed SARS-CoV-2 ORF10 protein expression (by IF, WB, and IP) in infected cells. Because ORF10 has no ortholog in SARS-CoV and ORF10 protein was previously predicted not to be expressed in infected cells [17], detection of ORF10 protein expression confirms that this ORF is a point of notable genomic divergence from SARS-CoV and could therefore be a determinant of the divergent transmissibility/pathology caused by SARS-CoV-2.

Antiviral drug screening and validation of FDA-approved drugs for repurposing to treat SARS-CoV-2 infection are key approaches in the search for effective antivirals. While modified cell lines are relatively easy to generate, they have been included in this toolkit as they are still useful tools for dissecting SARS-CoV-2 biology. For example, apilimod is a PIKfyve inhibitor and PIKfyve blockade potently inhibits SARS-CoV-2 infection in some cells [38,39], likely through interfering with endosomal trafficking [40]. Such an approach has been suggested to be unaffected by TMPRSS2 expression because PIKfyve inhibition could be independent and downstream of the proteases that prime the SARS-CoV-2 glycoprotein for fusion [41]. However, in contrast to this proposition, when exogenous TMPRSS2 was present on Vero E6- and A549-ACE2 cells, we show that apilimod was unable to protect cells from SARS-CoV-2 infection. We speculate that this is because the protease activity of TMPRSS2 might promote infection via direct fusion with the plasma membrane. This suggests that PIKfyve inhibition will only prove clinically effective if combined with effective protease inhibition. In addition, we demonstrated that modified Vero E6 and A549 cell systems were more permissive to SARS-CoV-2 infection and are ideal cell culture systems for isolating clinical viruses and investigating viral plaque phenotypes, which can vary considerably. Thus, these relatively simple tools are useful for dissecting the biology of SARS-CoV-2.

A key advantage of our COVID-19 coronavirus resource toolkit is that all antibodies (and corresponding proteins and cDNAs) listed, our lentiviral constructs (and stable Vero E6 and A549 cell lines), and our DNA-launched SARS-CoV-2 RG constructs are openly available through our not-for-profit web interface (antibodies, protein, rescue plasmids at https://mrcppu-covid.bio/; cell lines and virus strains have been deposited at the UK National Institute for Biological Standards and Controls; www.nibsc.org). By making this toolkit available to the global research community, these resources will fast-track investigation of many aspects of COVID-19 research, including drug discovery and vaccine development.

## Materials and methods

### DNA-launched SARS-CoV-2 plasmid construction

pCC1-4K-SARS-CoV-2-Wuhan-Hu1 (MT926410) and its derivatives, pCC1-4K-SARS-CoV-2-mCherry (MT926411), pCC1-4K-SARS-CoV-2-NLuc (MT926412), and pCC1-4K-SARS-CoV-2-ZsGreen (MW289908) were assembled from synthetic DNA fragments (Genscript). The clones were assembled from 5 synthetic DNAs assembled in a stepwise fashion using conventional restriction enzyme cloning.

### Rescue of infectious virus

A total of 3 μg of plasmid DNA (containing icDNA of SARS-CoV-2) and 3 μl of Lipofectamine LTX with 3 μl of PLUS reagent were used to transfect BHK-21 cells (ECACC) in 6-well plates. On the next 3 days posttransfection, supernatant was transferred to Vero E6 cultures in T25 flasks. Virus was allowed to propagate in Vero E6 cells for a further 4 days before harvesting the P0 stock. Infectious titres were enumerated by counting infected foci or via plaque assay. Rescued virus was used to infect Vero E6 cells in 96-well plates. For the Nanoluc assay, cells were lysed using a passive lysis buffer (Promega, cat#E1941) at 24 hpi. NanoLuc activity was determined using the Nano-Glo Luciferase Assay kit (Promega, Cat# N1130) and measured using a GloMax Luminometer (Promega).

### Modified cell lines

The pLV-EF1a-IRES-Hygro (Addgene plasmid #85134) and pLV-EF1a-IRES-Neo (Addgene, plasmid #85139) lentiviral vectors were modified to encode synthesised (Integrated DNA Technologies) ACE2 (GenBank NM_001371415.1) and TMPRSS2 (GenBank NM_005656). Lentiviral vectors were produced through transient transfection as described previously [42] and used to modify A549 cells (ATCC#CCL-185; generous gift from Prof. Ben Hale, validated by STR analysis (Eurofins)) and Vero E6 cells (ATCC#CRL-1586; generous gift of Prof. Michele Bouloy) that were cultured and transduced under standard culture conditions.

### Isolation of SARS-CoV-2 viruses from clinical samples

Sputum and BAL residual clinical samples from SARS-CoV-2-infected individuals were obtained for culture and used to inoculate Vero E6 or VAT cells in T25 flasks. Samples were harvested between 48 and 96 h postinfection, depending on the severity of CPE. Viral titers were determined by plaque assay.

### Sequencing and metagenomics of isolated SARS-CoV-2 viruses

To determine the sequence of CVR-GLA-1, CVR-GLA-2, and CVR-GLA-3 isolated from clinical samples (CVR837, CVR2224, CVR3899_BAL) and the RG-rescued SARS-CoV-2 viruses, RNA was extracted from culture supernatant using a hybrid Trizol-RNeasy protocol. Library was made from cDNA using Kapa LTP Library Preparation Kit for Illumina Platforms (Kapa Biosystems, cat# KK8232). The sequencing of the libraries was carried out on Illumina’s NextSeq 550 System (Illumina, cat# SY-415-1002).

### Plaque assays

Monolayers of A549 or Vero E6 cells and their ACE2/TMPRSS2 derivatives were prepared by seeding 2.5 × 10^5^ cells/well in 12-well plates, and plaque assays were executed using standard conditions. Briefly, a 1-ml overlay of MEM/0.6% Avicel RC-591/2% FCS was used, and fixed plates were stained with Coomassie Blue staining solution (0.1% Coomassie Brilliant Blue R-250/45% methanol/10% acetic acid) before scanning using a photo scanner (Epson Expression 1680 Pro).

### Well-clearance/monolayer integrity CPE assay

Cells were seeded in optical 96-well plates (Perkin Elmer CellCarrier-96 Ultra Microplates Cat# 6055302) The following day, the cells were incubated with 10 μM to 20 nM of the various compounds. The cells were then mock infected or infected with a predetermined dose of SARS-CoV-2 that would cause substantial CPE by 72 h postinfection. Fixed plates were stained (0.1% Coomassie Brilliant Blue R-250/45% methanol/10% acetic acid) and scanned using a Celigo imaging cytometer (Nexcelom), allowing monolayer integrity to be quantified (to facilitate quantification of well-clearance to assess virus growth and toxicity). Dead cell protease activity was measured in the same plates using the CytoTox-Glo Cytotoxicity Assay (Promega) in accordance with the manufacturer’s instructions.

### Generation of coronavirus antibodies

N-terminal GST fusion proteins for each coronavirus protein were used as antigens, and sheep were immunised with each antigen, followed by up to 5 further injections 28 days apart, with bleeds performed 7 days after each inoculation. Antibodies were subsequently affinity purified from serum using N-terminal MBP-tagged recombinant protein. All antibodies (and associated proteins and cDNA clones) generated in this paper are openly available at: https://mrcppu-covid.bio/.

### Ethics statement

This project falls under the scope and authority of project licence P134D890E, issued by the UK Home Office in accordance with the Animals (Scientific Procedures) Act 1986. This licence will stay in force, unless revoked earlier, until 2 April 2024. Prior to submission to the Home Office, the application for the licence was scrutinised by the University of Dundee Ethical Review Committee, which approved the proposal at its meeting in March 2019. The Committee (now termed the Welfare and Ethical Use of Animals Committee) is a subcommittee of the University Court, its highest governing body. All projects undertaken under the licence are reviewed in terms of scientific rigor and possible alternative methods to avoid animal use, as well as AWERB in terms of welfare.

### Immunofluorescence

Cells infected with SARS-CoV-2 were fixed and permeabilised in 8% formaldehyde/1% Triton X-100 at designated time points postinfection. DAPI (Thermo Fisher Scientific, cat# P36395) was included in the mounting medium, and slides were stained with primary sheep anti-coronavirus antibodies (1:500) and a rabbit anti-sheep secondary antibody (Abcam cat# ab150182) at a 1:1000 dilution before imaging (Zeiss LSM 880 confocal microscope). Alternatively, fixed and permeabilised cells were labelled using a mouse anti-N (nucleocapsid) IgG antibody (SinoBiologicals; cat# 40143-MM08), which was detected using an AlexaFluor 488-conjugated goat anti-mouse secondary antibody (Life Technologies; cat# A28175). Cells were subsequently counterstained with DAPI (1 μg/ml) and mounted using ProLong Gold antifade (Life Technologies; cat# P36930). Sequential channels were acquired as z-stacks (1.2 μm step) using an Olympus FV3000 confocal microscope and processed using Imaris software (v 9.0.2; Bitplane).

For the enumeration of infected foci, the sheep anti-SARS-CoV-2 N antibody was used (at a 1:500 dilution) and visualised using donkey anti-sheep antibody (A11015, Thermo Fisher Scientific, 1:1000 dilution) prior to scanning (Celigo, Nexcelom).

### Immunoprecipitation

At 3 days postinfection (at MOI 0.1), cells were trypsinised and rinsed in ice cold PBS before being lysed in ice-cold lysis buffer [43]. IPs were set up overnight at 4°C with the antibody and protein lysate in a ratio of 3 μg of antibody per mg of protein lysate. The immunoprecipitated proteins were subjected to SDS-PAGE and then transferred to PVDF membranes (Merck Millipore, IPFL00010), which were then incubated with their respective biotinylated primary antibodies (0.5 to 1.0 mg/ml). Bands were visualised (LI-COR Odyssey CLx imaging system) using labelled streptavidin (DyLight 680/800, Thermo Fisher Scientific, 21848/21851).

### Western blotting

Briefly, sonicated cell lysates were resolved using Novex 4% to 12% acrylamide gels (Life Technologies) before wet-transfer onto nitrocellulose membranes (Sigma Aldrich, cat# GE10600003), after which membranes were probed with the indicated primary antibody (full list of antibodies and their availability found in S2 Table) in SEA BLOCK (Thermo Scientific, cat# 37527), unless otherwise specified. Bands were visualised using DyLight-labelled secondary antibodies (Thermo Fisher Scientific) prior to scanning with a Li-Cor Odyssey CLx imaging system.

### Materials availability

All sheep CoV antibodies (and corresponding proteins and cDNAs) listed, lentiviral constructs (and stable Vero E6 and A549 cell lines), and DNA-launched SARS-CoV-2 RG constructs are openly available through a not-for-profit web interface (antibodies, proteins, rescue plasmids at https://mrcppu-covid.bio/; cell lines and virus strains have been deposited at the UK National Institute for Biological Standards and Controls; www.nibsc.org).

## Supporting information

S1 Fig Sequencing coverage of the sequenced rescue plasmids and rescued viruses. (A) Summary plots of the number of reads mapping to the passaged plasmids from Fig 1B. (B) Summary plots of the number of reads mapping to the SARS-CoV-2-mCherry, NLuc, and ZsGreen rescue plasmids from the sequenced plasmid and the sequenced rescued virus (for each rescue system). NLuc, Nanoluciferase; SARS-CoV-2, Severe Acute Respiratory Syndrome Coronavirus 2; wt, wild type.
(PDF)

S2 Fig Validation of the antibodies generated in this study by indirect immunofluorescence. (A, B) As in Fig 2C, Vero E6 cells were uninfected (mock) or infected with SARS-CoV-2 England-02 at an MOI of 0.1 for 48 h before fixation/permeabilisation. Cells were stained with primary sheep antibodies diluted at 1:500 and secondary rabbit anti-sheep Alexa 555 diluted at 1:1,000. MOI, multiplicity of infection; SARS-CoV-2, Severe Acute Respiratory Syndrome Coronavirus 2.
(PDF)

S3 Fig Validation of the antibody reactivity by western blotting. (A, B) As in Fig 2D, Vero E6 cells were uninfected (mock) or infected with SARS-CoV-2 England-02 at an MOI of 0.1 or 1 (as indicated) for 72 h prior to WB of whole cell lysates. Arrows indicate the band of interest. MOI, multiplicity of infection; SARS-CoV-2, Severe Acute Respiratory Syndrome Coronavirus 2; WB, western blotting.
(PDF)

S4 Fig Cross-reactivity of coronavirus nucleocapsid (N) and envelope (E) proteins. (A) WB analysis of cross-reactivity of N-specific antibodies to SARS-CoV, MERS-CoV, HCoV 229E, and HCoV OC43 to the N protein of SARS-CoV-2. Vero E6 cells were mock infected (mock) or infected with SARS-CoV-2 England-02 at an MOI of 0.1 or 1 for 72 h and probed as in S3 Fig. (B) A comparison of IP results for the N proteins from SARS-CoV, MERS-CoV, HCoV 229E, and HCoV OC43. As in Fig 2E, Vero E6 cells were uninfected (mock) or infected with SARS-CoV-2 England-02 at an MOI of 0.1 for 3 days, followed by lysis, IP, and blotting with the indicated N protein. (C) As in (B) but for the E proteins of SARS-CoV and MERS-CoV. (D) WB analysis as in (A) but using MERS-CoV and SARS-CoV E antibodies. HCoV, human coronavirus; IB, immunoblotting; IP, immunoprecipitation; MERS-CoV, Middle East Respiratory Syndrome Coronavirus; MOI, multiplicity of infection; SARS-CoV-2, Severe Acute Respiratory Syndrome Coronavirus 2; WB, western blotting.
(PDF)

S5 Fig Validation of antibody reactivity by immunoprecipitation. (A, B) As in Fig 2E, Vero E6 cells were uninfected (mock) or infected with SARS-CoV-2 England-02 at an MOI of 0.1 for 3 days. The cells were then lysed, and the viral proteins immunoprecipitated and detected by WB using the indicated antibodies. No specific bands were present in the infected cells for the SARS-CoV-2 E antibody. IB, immunobloting; IP, immunoprecipitation; MOI, multiplicity of infection; SARS-CoV-2, Severe Acute Respiratory Syndrome Coronavirus 2; WB, western blotting.
(PDF)

S6 Fig A demonstration of the utility of the modified AA and AAT cell lines for conducting phenotypic assays. (A) Anti-SARS-CoV-2 dose response curves of a panel of compounds using the well-clearance assay in AA cells and AAT cells (Fig 4K–4N) multiplexed with a dead cell protease toxicity assay. The mean and standard error from 4 replicate experiments is plotted. The apilimod panels (top right) plot the corresponding toxicity data to the data included in Fig 4L. The labels for CsA and HCQ are abbreviated. (B) As in panel A, an extended dose response of camostat in AAT cells is shown. The data underlying S6A and S6B Fig may be found in S1 Data. AA, A549-ACE2; AAT, A549-ACE2-TMPRSS2; CsA, cyclosporine A; HCQ, hydroxychloroquine; SARS-CoV-2, Severe Acute Respiratory Syndrome Coronavirus 2.
(PDF)

S1 Text Extended Materials and methods. (PDF)

S1 Table Sequence variation in passaged SARS-CoV-2-mCherry and SARS-CoV-2 CVR-GLA-1. (XLSX)

S2 Table Reagents and resources. (PDF)

S1 Data Underlying data. (XLSX)

S1 Raw Images Raw data image files. (PDF)

## Abbreviations

AA: A549-ACE2
AAT: A549-ACE2-TMPRSS2
ACE2: angiotensin-converting enzyme 2
BAC: bacterial artificial chromosome
BAL: bronchoalveolar lavage
CMV: cytomegalovirus
co-IP: co-immunoprecipitation
COVID-19: Coronavirus Disease 2019
CPE: cytopathic effect
FMDV: foot-and-mouth disease virus
GST: glutathione S-transferase
HCoV: human coronavirus
HDV: hepatitis delta virus
icDNA: infectious cDNA
IF: immunofluorescence
IgG: immunoglobulin G
IP: immunoprecipitation
MBP: maltose-binding protein
MERS-CoV: Middle East Respiratory Syndrome Coronavirus
MOI: multiplicity of infection
NLuc: Nanoluciferase
nsp: nonstructural proteins
RG: reverse genetics
SARS-CoV-2: Severe Acute Respiratory Syndrome Coronavirus 2
SV40: Simian Virus 40 terminator
VA: Vero E6-ACE2
VAT: Vero E6-ACE2-TMPRSS2
VT: Vero E6-TMPRSS2
WB: western blotting
wt: wild-type
YAC: yeast artificial chromosome
