# Supplementary figures and images for: A plasmid DNA-launched SARS-CoV-2 reverse genetics system and coronavirus toolkit for COVID-19 research
Source: PLoS Biol. 2021 Feb 25;19(2):e3001091. doi: 10.1371/journal.pbio.3001091 (PMC7906417; doi:10.1371/journal.pbio.3001091)

**A**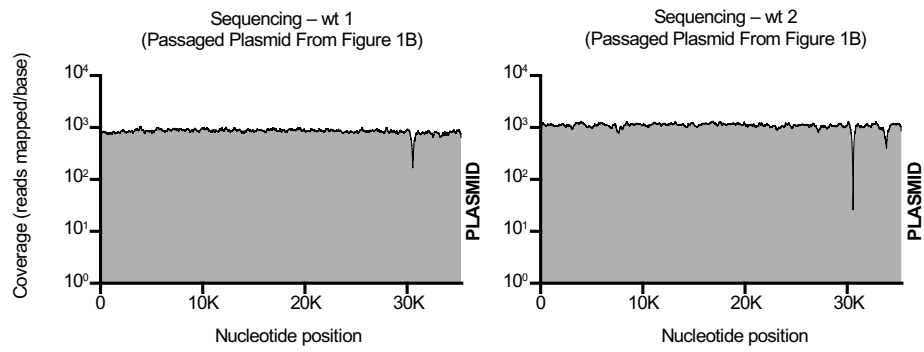**B**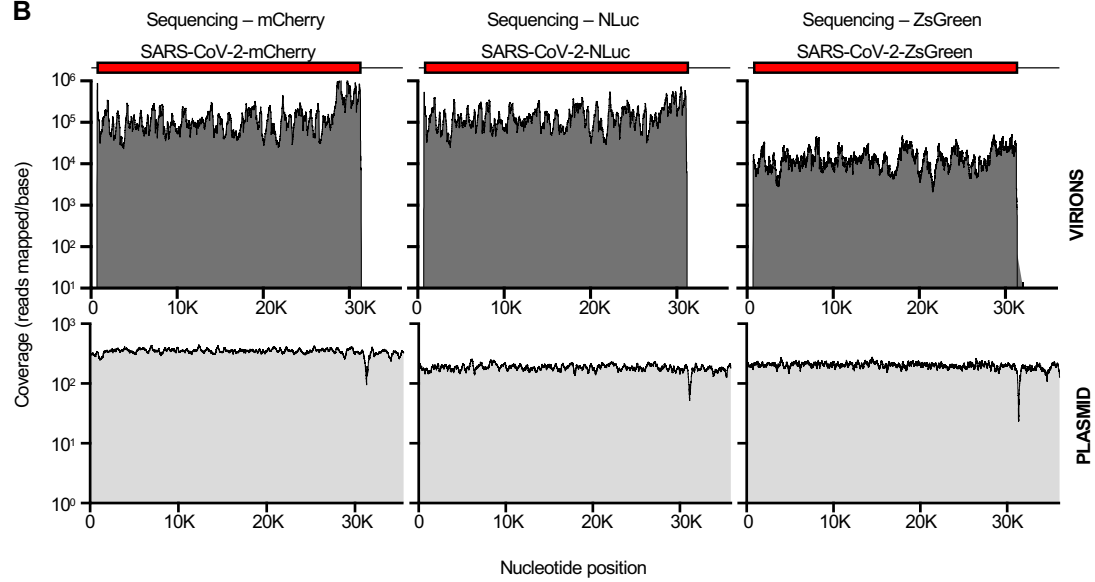

Supplement: S1 Fig — (A) Summary plots of the number of reads mapping to the passaged plasmids from Fig 1B. (B) Summary plots of the number of reads mapping to the SARS-CoV-2-mCherry, NLuc, and ZsGreen rescue plasmids from the sequenced plasmid and the sequenced rescued virus (for each rescue system). NLuc, Nanoluciferase; SARS-CoV-2, Severe Acute Respiratory Syndrome Coronavirus 2; wt, wild type. (PDF) [file pbio.3001091.s001.pdf]

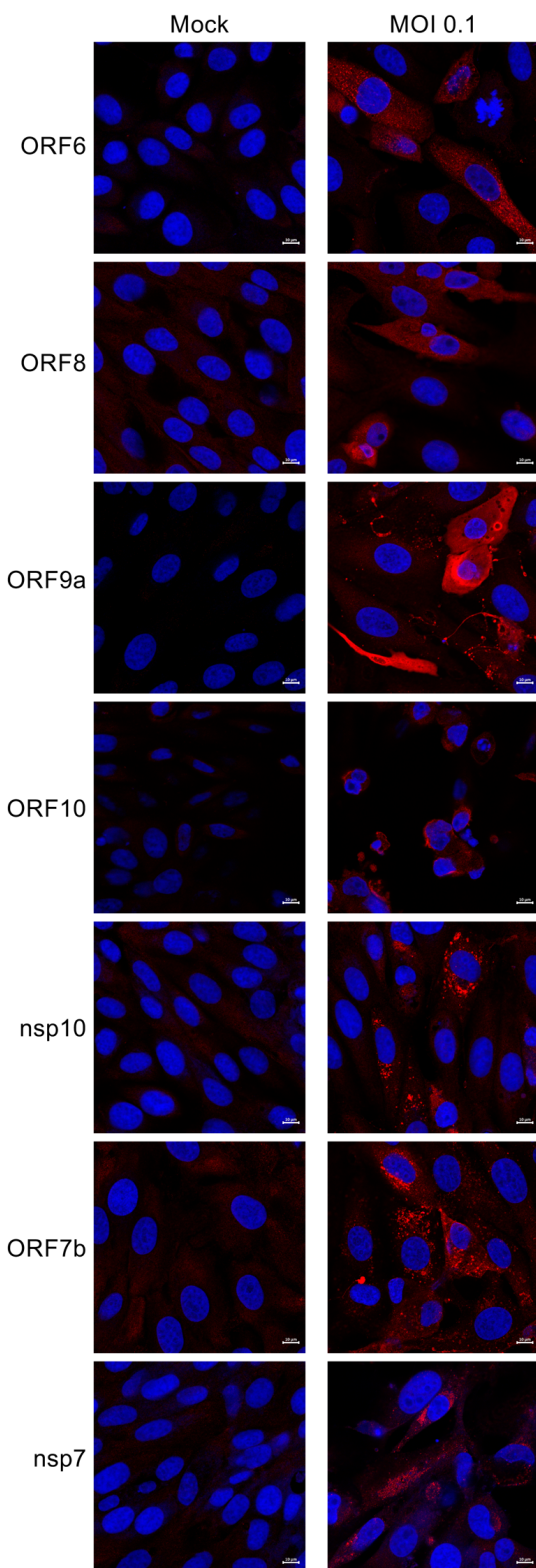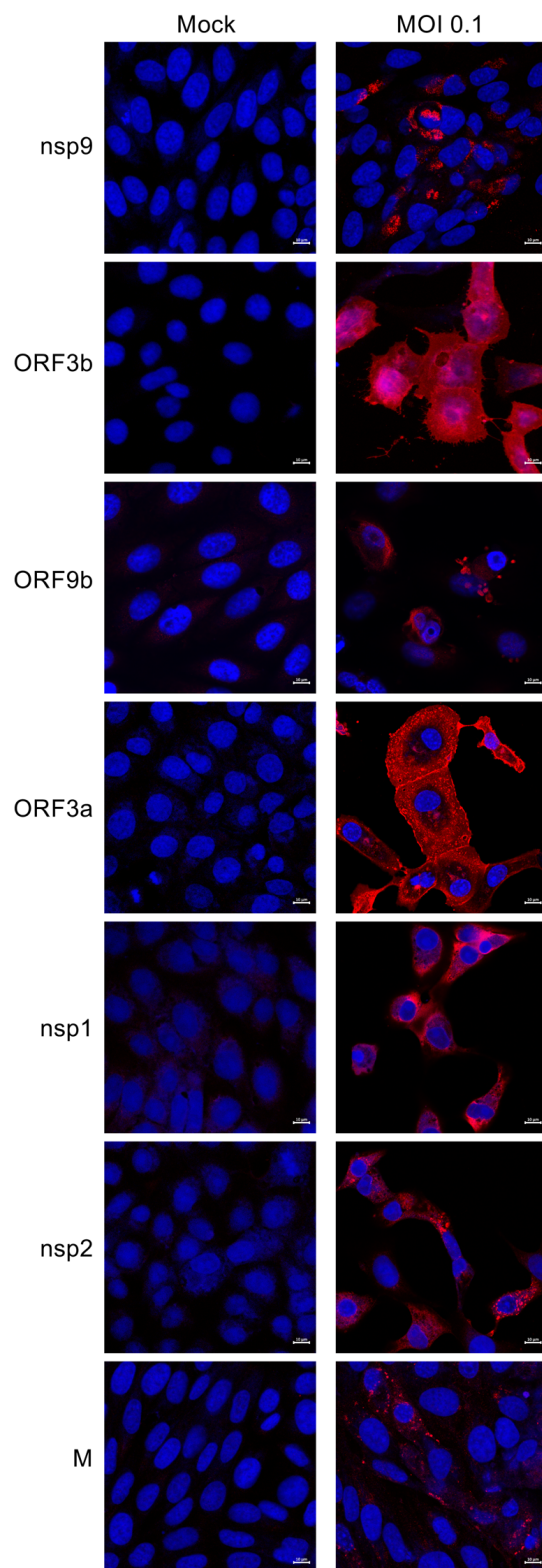

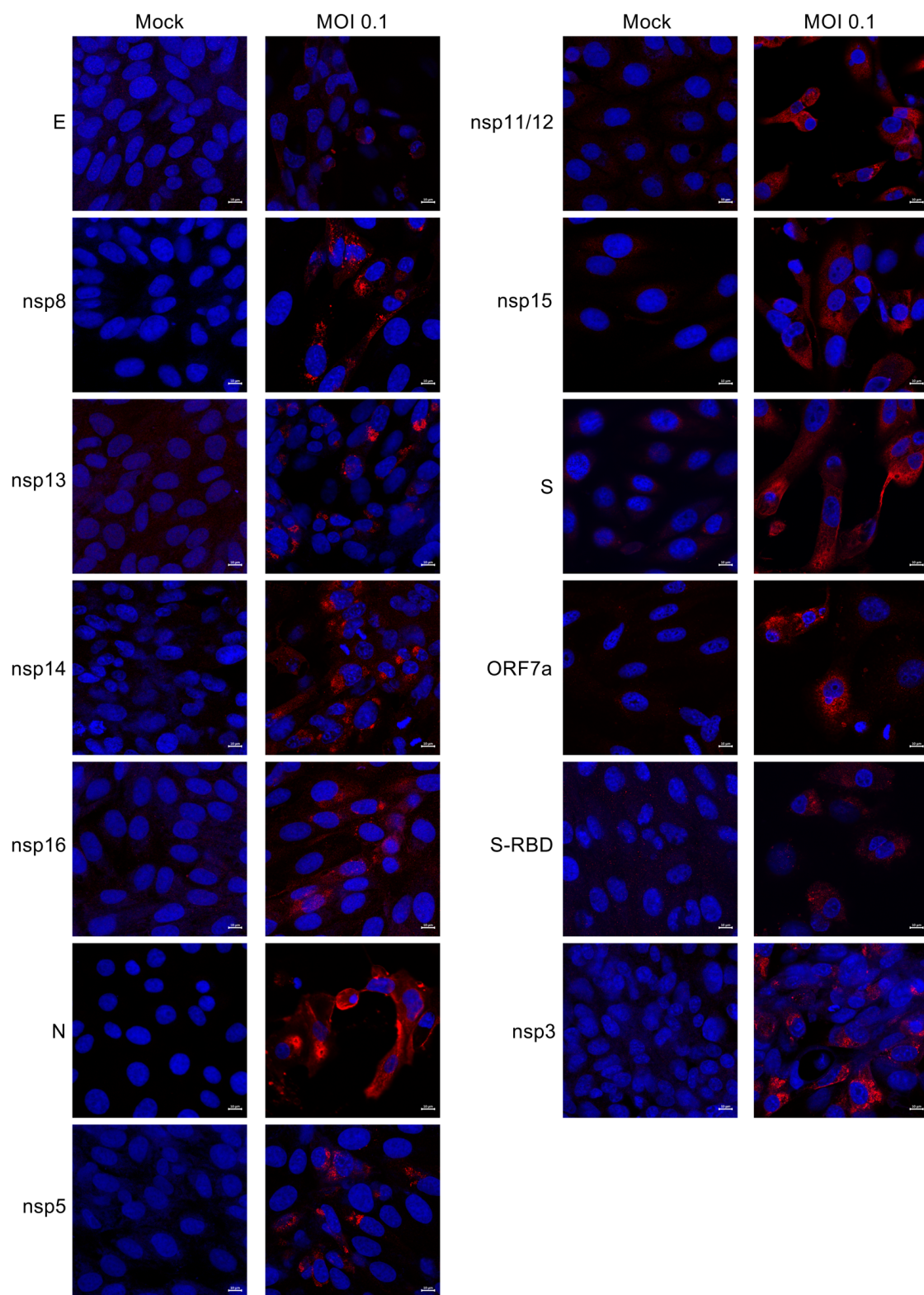

Supplement: S2 Fig — (A, B) As in Fig 2C, Vero E6 cells were uninfected (mock) or infected with SARS-CoV-2 England-02 at an MOI of 0.1 for 48 h before fixation/permeabilisation. Cells were stained with primary sheep antibodies diluted at 1:500 and secondary rabbit anti-sheep Alexa 555 diluted at 1:1,000. MOI, multiplicity of infection; SARS-CoV-2, Severe Acute Respiratory Syndrome Coronavirus 2. (PDF) [file pbio.3001091.s002.pdf]

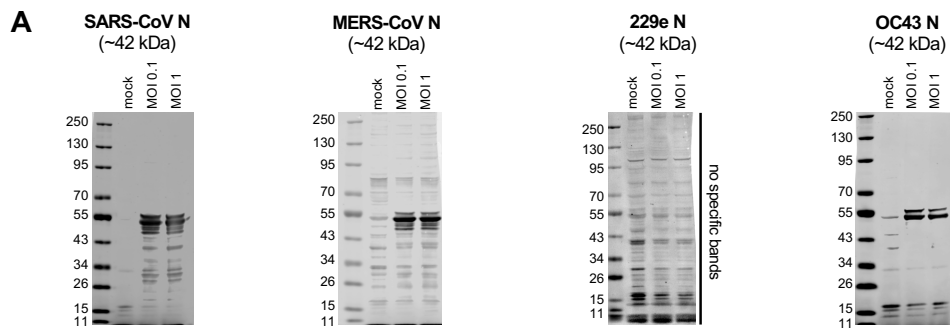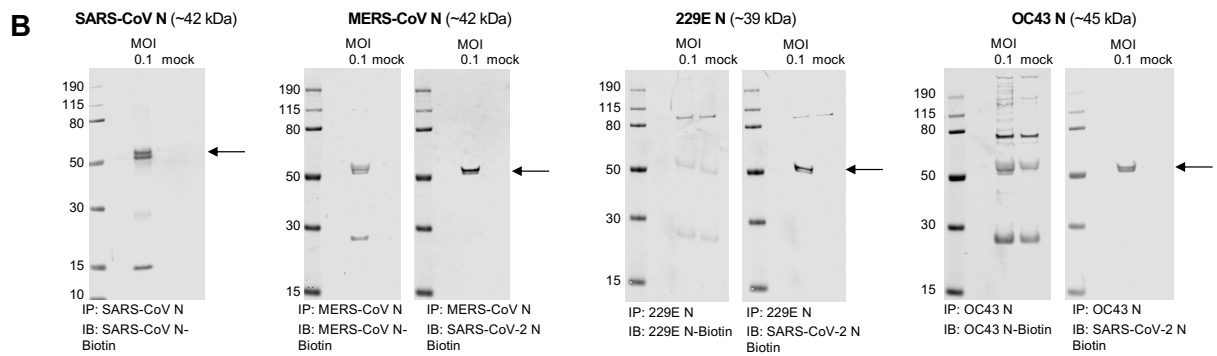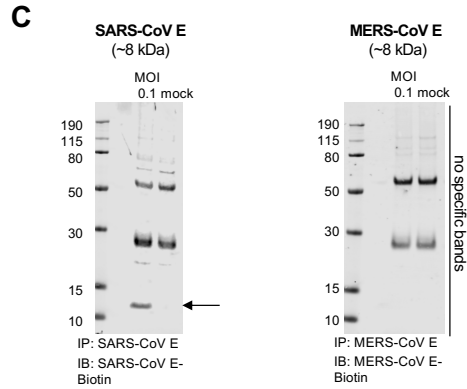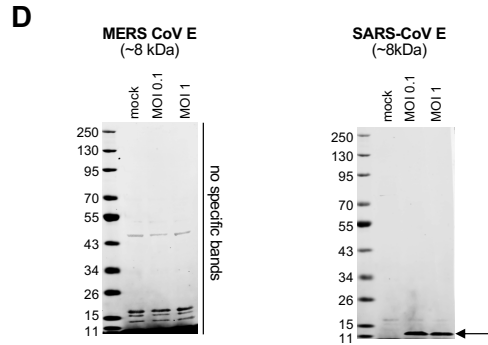

Supplement: S4 Fig — (A) WB analysis of cross-reactivity of N-specific antibodies to SARS-CoV, MERS-CoV, HCoV 229E, and HCoV OC43 to the N protein of SARS-CoV-2. Vero E6 cells were mock infected (mock) or infected with SARS-CoV-2 England-02 at an MOI of 0.1 or 1 for 72 h and probed as in S3 Fig. (B) A comparison of IP results for the N proteins from SARS-CoV, MERS-CoV, HCoV 229E, and HCoV OC43. As in Fig 2E, Vero E6 cells were uninfected (mock) or infected with SARS-CoV-2 England-02 at an MOI of 0.1 for 3 days, followed by lysis, IP, and blotting with the indicated N protein. (C) As in (B) but for the E proteins of SARS-CoV and MERS-CoV. (D) WB analysis as in (A) but using MERS-CoV and SARS-CoV E antibodies. HCoV, human coronavirus; IB, immunoblotting; IP, immunoprecipitation; MERS-CoV, Middle East Respiratory Syndrome Coronavirus; MOI, multiplicity of infection; SARS-CoV-2, Severe Acute Respiratory Syndrome Coronavirus 2; WB, western blotting. (PDF) [file pbio.3001091.s004.pdf]

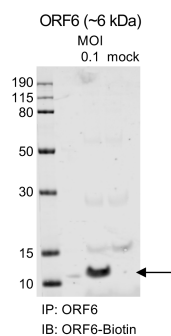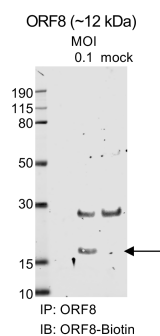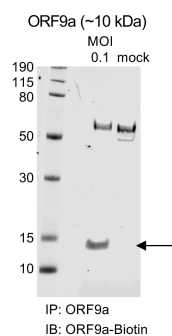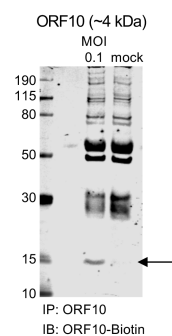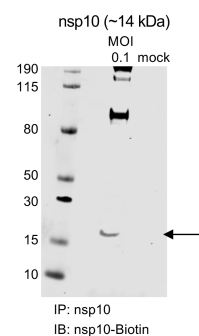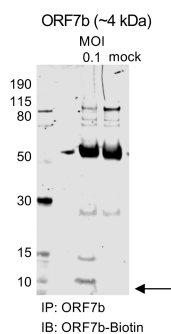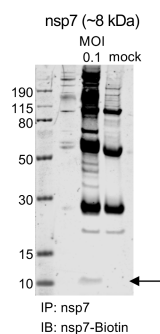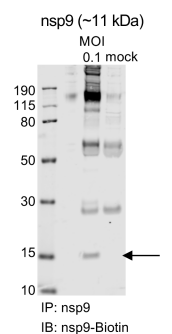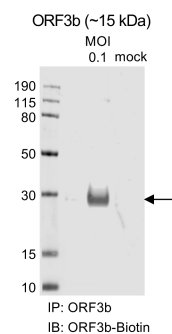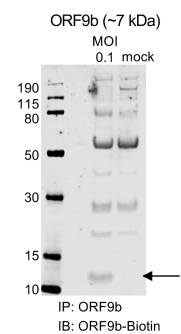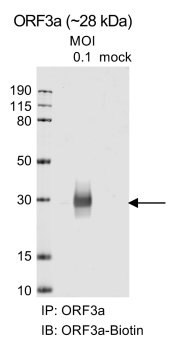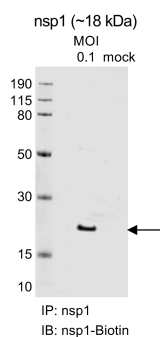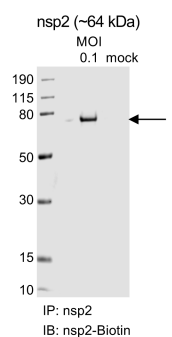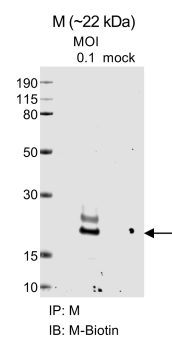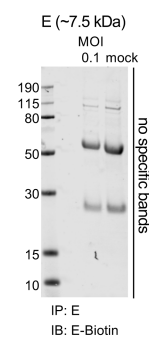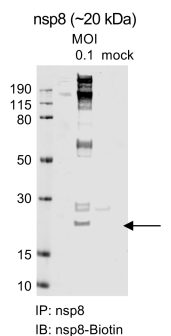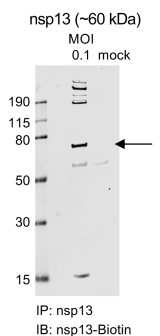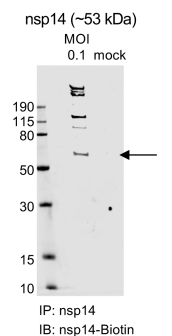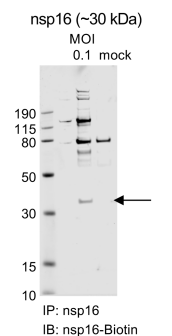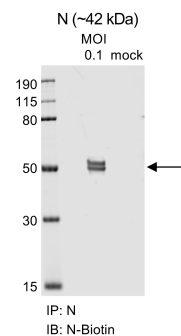

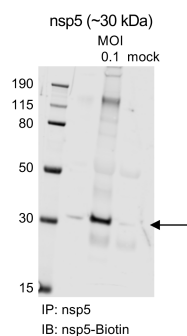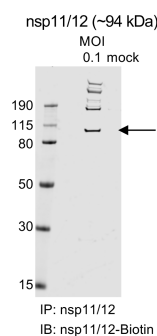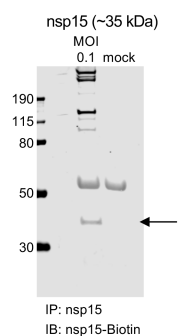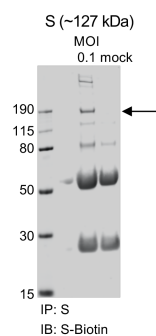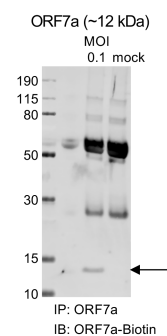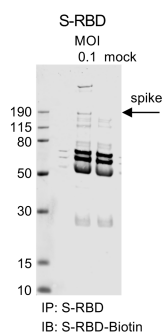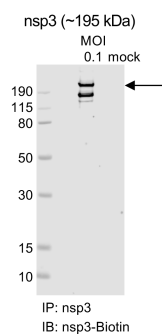

Supplement: S5 Fig — (A, B) As in Fig 2E, Vero E6 cells were uninfected (mock) or infected with SARS-CoV-2 England-02 at an MOI of 0.1 for 3 days. The cells were then lysed, and the viral proteins immunoprecipitated and detected by WB using the indicated antibodies. No specific bands were present in the infected cells for the SARS-CoV-2 E antibody. IB, immunobloting; IP, immunoprecipitation; MOI, multiplicity of infection; SARS-CoV-2, Severe Acute Respiratory Syndrome Coronavirus 2; WB, western blotting. (PDF) [file pbio.3001091.s005.pdf]

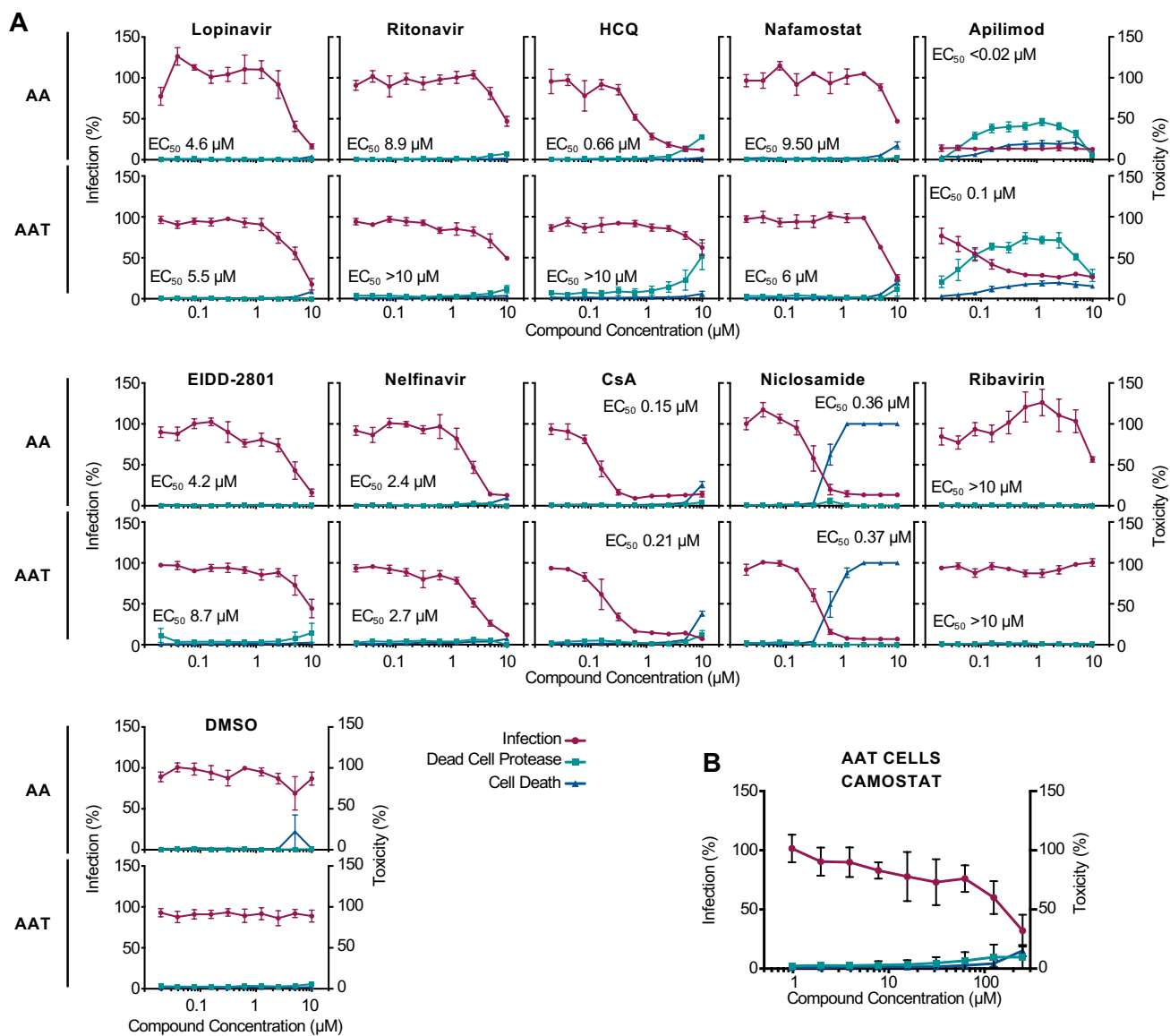

Supplement: S6 Fig — (A) Anti-SARS-CoV-2 dose response curves of a panel of compounds using the well-clearance assay in AA cells and AAT cells (Fig 4K–4N) multiplexed with a dead cell protease toxicity assay. The mean and standard error from 4 replicate experiments is plotted. The apilimod panels (top right) plot the corresponding toxicity data to the data included in Fig 4L. The labels for CsA and HCQ are abbreviated. (B) As in panel A, an extended dose response of camostat in AAT cells is shown. The data underlying S6A and S6B Fig may be found in S1 Data. AA, A549-ACE2; AAT, A549-ACE2-TMPRSS2; CsA, cyclosporine A; HCQ, hydroxychloroquine; SARS-CoV-2, Severe Acute Respiratory Syndrome Coronavirus 2. (PDF) [file pbio.3001091.s006.pdf]
