# Supplementary material for: A plasmid DNA-launched SARS-CoV-2 reverse genetics system and coronavirus toolkit for COVID-19 research
Source: PLoS Biol. 2021 Feb 25;19(2):e3001091. doi: 10.1371/journal.pbio.3001091 (PMC7906417; doi:10.1371/journal.pbio.3001091)
Supplement: S3 Fig — (A, B) As in Fig 2D, Vero E6 cells were uninfected (mock) or infected with SARS-CoV-2 England-02 at an MOI of 0.1 or 1 (as indicated) for 72 h prior to WB of whole cell lysates. Arrows indicate the band of interest. MOI, multiplicity of infection; SARS-CoV-2, Severe Acute Respiratory Syndrome Coronavirus 2; WB, western blotting. (PDF) [file pbio.3001091.s003.pdf]

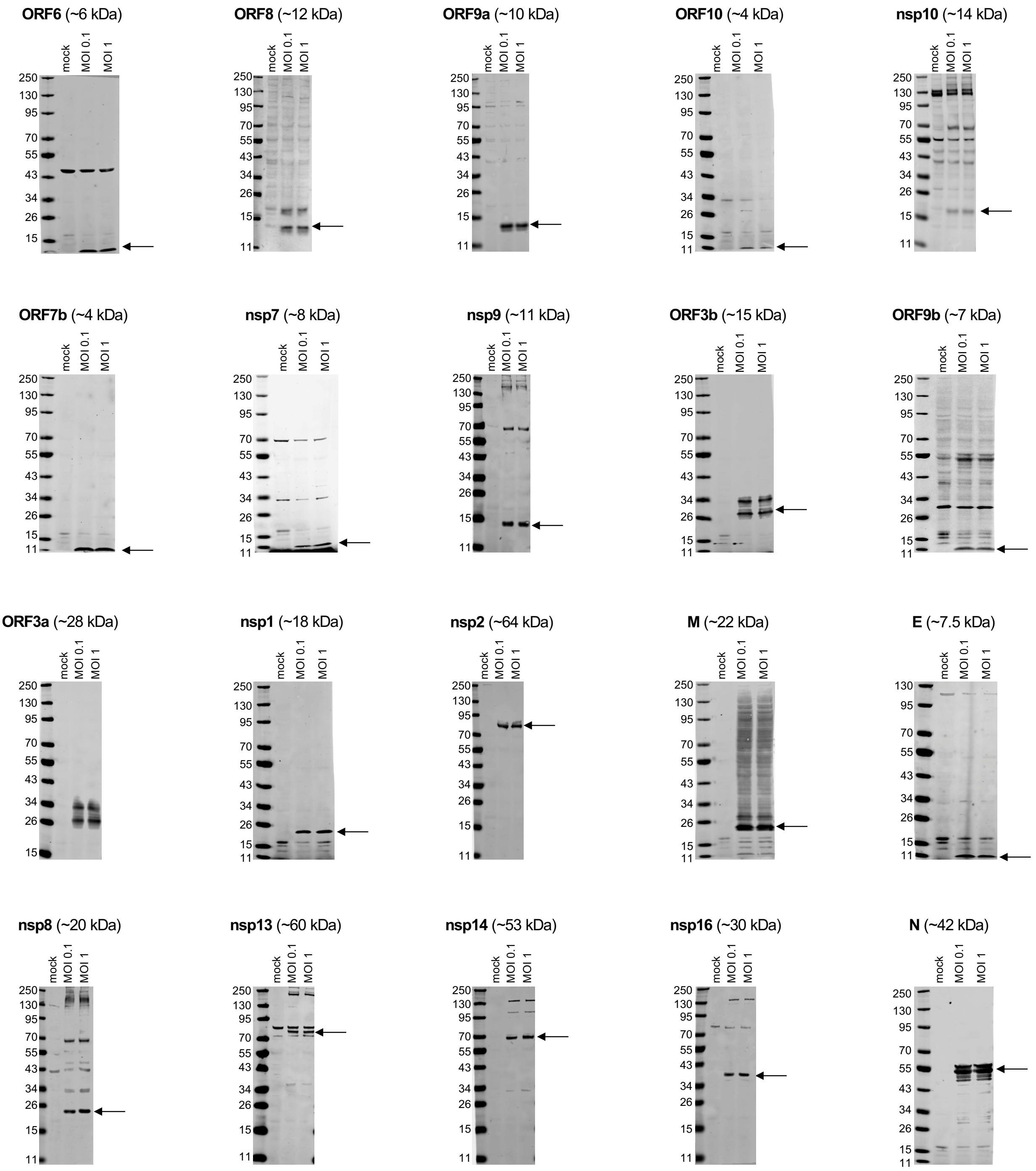
ORF6 (~6 kDa)
mock
MOI 0.1
MOI 1
250 130 95 70 55 43 34 26 15
ORF8 (~12 kDa)
mock
MOI 0.1
MOI 1
250 130 95 70 55 43 34 26 15 11
ORF9a (~10 kDa)
mock
MOI 0.1
MOI 1
250 130 95 70 55 43 34 26 15 11
ORF10 (~4 kDa)
mock
MOI 0.1
MOI 1
250 130 95 70 55 43 34 26 15 11
nsp10 (~14 kDa)
mock
MOI 0.1
MOI 1
250 130 95 70 55 43 34 26 15 11
ORF7b (~4 kDa)
mock
MOI 0.1
MOI 1
250 130 95 70 55 43 34 26 15 11
nsp7 (~8 kDa)
mock
MOI 0.1
MOI 1
250 130 95 70 55 43 34 26 15 11
nsp9 (~11 kDa)
mock
MOI 0.1
MOI 1
250 130 95 70 55 43 34 26 15 11
ORF3b (~15 kDa)
mock
MOI 0.1
MOI 1
250 130 95 70 55 43 34 26 15 11
ORF9b (~7 kDa)
mock
MOI 0.1
MOI 1
250 130 95 70 55 43 34 26 15 11
ORF3a (~28 kDa)
mock
MOI 0.1
MOI 1
250 130 95 70 55 43 34 26 15
nsp1 (~18 kDa)
mock
MOI 0.1
MOI 1
250 130 95 70 55 43 34 26 15 11
nsp2 (~64 kDa)
mock
MOI 0.1
MOI 1
250 130 95 70 55 43 34 26 15 11
M (~22 kDa)
mock
MOI 0.1
MOI 1
250 130 95 70 55 43 34 26 15 11
E (~7.5 kDa)
mock
MOI 0.1
MOI 1
130 95 70 55 43 34 26 15 11
nsp8 (~20 kDa)
mock
MOI 0.1
MOI 1
250 130 95 70 55 43 34 26 15 11
nsp13 (~60 kDa)
mock
MOI 0.1
MOI 1
250 130 95 70 55 43 34 26 15 11
nsp14 (~53 kDa)
mock
MOI 0.1
MOI 1
250 130 95 70 55 43 34 26 15 11
nsp16 (~30 kDa)
mock
MOI 0.1
MOI 1
250 130 95 70 55 43 34 26 15 11
N (~42 kDa)
mock
MOI 0.1
MOI 1
250 130 95 70 55 43 34 26 15 11

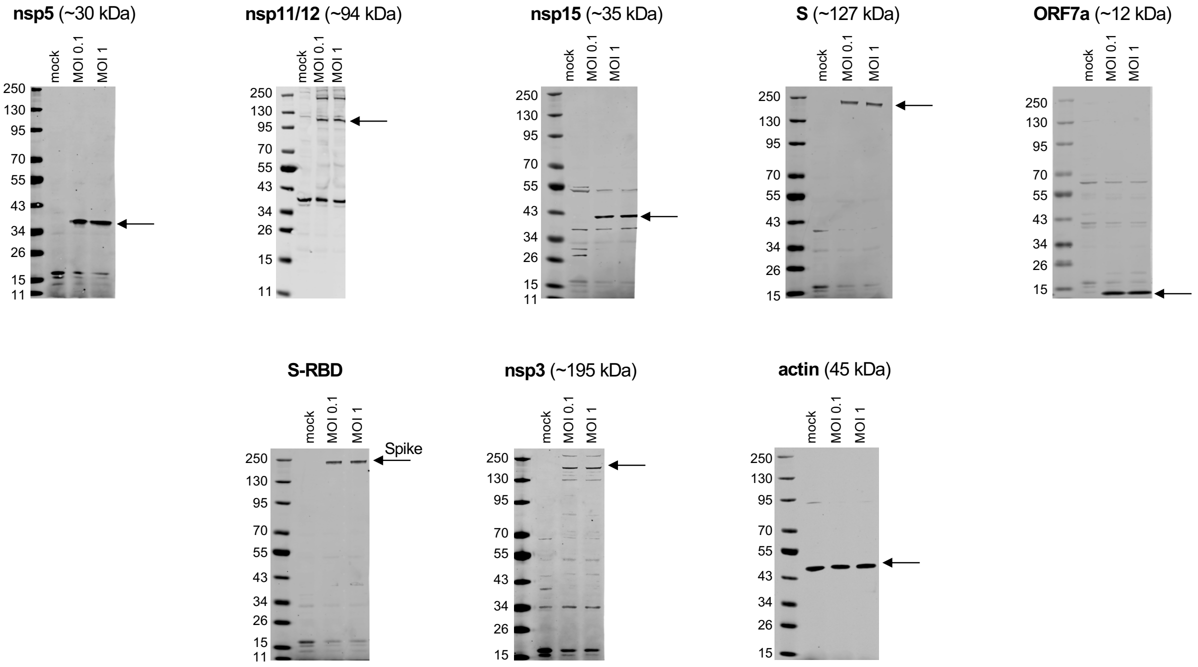
nsp5 (~30 kDa)
nsp11/12 (~94 kDa)
nsp15 (~35 kDa)
S (~127 kDa)
ORF7a (~12 kDa)
S-RBD
nsp3 (~195 kDa)
actin (45 kDa)
mock
MOI 0.1
MOI 1
Spike
250
130
95
70
55
43
34
26
15
11
