# Supplementary material for: A plasmid DNA-launched SARS-CoV-2 reverse genetics system and coronavirus toolkit for COVID-19 research
Source: PLoS Biol. 2021 Feb 25;19(2):e3001091. doi: 10.1371/journal.pbio.3001091 (PMC7906417; doi:10.1371/journal.pbio.3001091)
Supplement: S1 Text — (PDF) [file pbio.3001091.s007.pdf]

S1 Text. Extended Materials and Methods

Design of reverse genetics (RG) SARS-CoV-2 clone design. pCC1-4K-SARS-CoV-2, pCC1-4K-SARS-CoV-2-mCherry and pCC1-4K-SARS-CoV-2-NLuc were made from synthetic DNA fragments (Genscript). Similarly, pCC1-4K-SARS-CoV-2-ZsGreen was also made from a synthetic *Bam*HI-*Xho*I fragment (Bio Basic). pCC1-4K-SARS-CoV-2 was assembled from five synthetic DNAs (**Figure 1**) using restriction endonuclease based cloning procedures. In order to increase the cloning efficiency and to monitor for possible assembly errors the assembly was performed using stepwise protocol (FR1+FR5 →FR15+FR4 → FR145+FR2 →FR1245+FR3 → full-length clone). The assembly of pCC1-4K-SARS-CoV-2-mCherry and pCC1-4K-SARS-CoV-2-NLuc was performed the same way except the marker-gene containing fragment was added to FR1 prior to adding other fragments. The exception was pCC1-4K-SARS-CoV-2-mCherry where the BamHI-XhoI fragment was inserted in the BamHI-XhoI digested wt pCC1-4K-SARS-CoV-2 backbone. The pCC1-4K based plasmids were propagated in TransforMax™ EPI300™ Electrocompetent *E. coli* which were transformed using electroporation (BioRad Gene Pulser Xcel) and grown in Tryptic Soy Broth/Agar (Becton Dickinson) supplemented chloramphenicol at 12.5 μg/ml. Purification of plasmids used for transfection was performed as follows: 200 ml overnight bacterial culture was diluted 1:5 with growth media and induced by adding D-arabinose at final concentration 0.1%. Culture was incubated at 37°C for 5 hrs before bacteria were lysed; endotoxin free DNA was purified using Nucleobond Xtra Midi EF kit (Machery Nagel, 740420.50).

Lentiviral vector design. The pLV-EF1a-IRES-Hygro (Addgene plasmid #85134) and pLV-EF1a-IRES-Neo (Addgene plasmid #85139) lentiviral vectors were modified to include *Sfi*I sites flanking the transgene by sub-cloning the TagRFP ORF with flanking *Sfi*I sites between the unique *BamH*I and *EcoR*I restriction sites using PCR (primer pair AW177-*BamH*I-*Sfi*I-RFP-F' and AW178-EcoRI-*Sfi*I-RFP-R', described in S2 Table). To make pLV-EF1a-IRES-Hygro-ACE2 (also referred to as pLV-ACE2 or pLV-Hygro-ACE2), a DNA sequence of the *Homo sapiens* angiotensin I converting enzyme II (ACE2) ORF (GenBank NM_001371415.1) was chemically synthesised in pEX-A258 (Eurofins) with corresponding flanking *Sfi*I sites. The *Sfi*I fragment was then sub-cloned into the modified pLV-EF1a-IRES-Hygro plasmid. For *Homo sapiens* transmembrane serine protease 2 (TMPRSS2) the DNA sequence of the ORF (GenBank NM_005656) was synthesised as a gBlock (Integrated DNA Technologies) with flanking *Sfi*I sites. The product was amplified by PCR (primers MT_313 HsTMPRSS2 5' *Sfi*I and MT_314 HsTMPRSS2 3' *Sfi*I described in S2 Table) and the *Sfi*I fragment was subcloned into modified pLV-EF1a-IRES-Neo. The ACE2 and TMPRSS2 ORFs in pLV-EF1a-IRES-Hygro-ACE2 (also referred to as pLV-ACE2 or pLV-Hygro-ACE2) and pLV-EF1a-IRES-Neo-TMPRSS2 (also referred to as pLV-TMPRSS2 or pLV-Neo-TMPRSS2), respectively, were sequence verified by Sanger sequencing (Eurofins). The GagPol expression plasmid pNLGP and the VSV glycoprotein expression plasmid pVSV-G have been described previously (Rihn *et al.*, 2019). The HIV-1 LNEIE capsid in the pNLGP-CapNM background (Soll *et al.*, 2013) was a kind gift of Prof. Paul Bieniasz.

Cells and generation of cell lines expressing exogenous ACE2 and TMPRSS2. A549 (ATCC #CCL-185; generous gift from Prof. Ben Hale) and Vero E6 cells (generous gift of Prof. Michele Bouloy) were maintained in Dulbecco's modified Eagle's medium (DMEM) supplemented with 9% fetal calf serum (FCS) and 10 μg/ml gentamicin. BHK-21 cells (ATCC #CCL-10, purchased from ATCC, Bethesda, MD) were maintained in DMEM supplemented with 5% fetal calf serum (FCS; Life Technologies).

Lentiviral vectors pseudotyped with the VSV glycoprotein were produced by transient transfection of HEK 293T cells with 5 μg of lentivirus vector plasmid, 5 μg of GagPol expression plasmid, and 1 μg of VSV-G expression plasmid. The supernatant containing the lentiviral vector was passed through a 0.2 μm pore size filter and used to transduce relevant cells. A549 and Vero E6 cells were transduced with LV-Hygro-ACE2 and/or LV-Neo-TMPRSS2 lentiviral vectors (LNEIE capsid was used for transduction of Vero E6 with the LV-Neo-TMPRSS2 lentiviral vector) and selected with either hygromycin B (200 μg/ml) or G418 (2 mg/ml) respectively. For dual ACE2- and TMPRSS2-overexpressing cells, hygromycin B-selected -ACE2 overexpressing bulk populations were subsequently transduced with LV-Neo-TMPRSS2 and selected with G418.

Isolation of SARS-CoV-2 viruses from clinical samples. Sputum and bronchoalveolar lavage (BAL) residual clinical samples from SARS-CoV-2-infected individuals were obtained for culture following informed consent (written) as part of the ISARIC4C study and sequencing was carried out at the CVR within the context of the COG-UK consortium (patient sample IDs: CVR837 sputum (GISAID accession:

EPI_ISL_461705) for CVR-GLA-1, CVR2224 sputum (GISAID accession: EPI_ISL_448167) for CVR-GLA-2, CVR3899_BAL (GISAID accession: EPI_ISL_490695) for CVR-GLA-3). Ethical approvals were granted for COG-UK (16/WS/0207NHS and 10/ S1402/33) and for ISARIC4C England and Wales (13/SC/0149), and Scotland (20/SS/0028). Specifically, Vero E6 or Vero E6-ACE2-TMPRSS2 cells were seeded at $3.5x10^5$ cells per T25 flask one day prior to inoculation. Upon arrival, clinical samples were resuspended in 1-2 ml of serum-free DMEM supplemented with 10 μg/ml gentamicin, 100 units/ml penicillin-streptomycin and 2.5 μg/ml amphotericinB. Up to 500 μl of the resuspended clinical sample was subsequently used for inoculation of the T25 flask in a volume of 5 ml DMEM supplemented with 2% FCS, 10 μg/ml gentamicin, 100 units/ml penicillin-streptomycin and 2.5 μg/ml amphotericinB. At 18 hrs post-inoculation, the medium was changed to remove residual sample debris. Daily observation of the cell monolayer for CPE was carried out. Samples were harvested between 48-96 hrs post-infection, depending on the severity of CPE. Supernatants were centrifuge clarified at 500 x g for 10 minutes at 4°C, aliquoted and stored at -80°C. Viral titers were determined by plaque assay. Subsequent passaging of clinical isolates was done on Vero E6 cells.

Sequencing of clinical isolates and rescued viruses. Extracted nucleic acid was incubated with DNAseI (Thermo Fisher, Part Number AM2222) for 5 minutes at 37°C. After DNAse treatment, the samples were purified using Agencourt RNA Clean AMPure XP Beads (Beckman Coulter, cat# A63987), following the manufacturer's guidelines, and quantified using the Qubit dsDNA HS Kit (Thermo Scientific, cat# Q32854). cDNA was synthesised using SuperScript III (Thermo Scientific, cat#

18080044) and NEBNext Ultra II Non-Directional RNA Second Strand Synthesis Module (New England Biolabs, cat# E6111L), as per the manufacturer's guidelines. CVR-GLA-1 was processed using the Illumina Nextera DNA Flex Kit (Illumina, cat# 20018704), following the manufacture's guideline and IDT for Illumina Nextera DNA Unique Dual Indexes (Illumina cat# 20027213). All the remaining samples were processed utilising the Kapa LTP Library Preparation Kit for Illumina Platforms (Kapa Biosystems, cat# KK8232). Briefly, the cDNA was end repaired and the protocol followed through to adapter ligation. At this stage the samples were uniquely indexed using the NEBNext Multiplex Oligos for Illumina 96 Unique Dual Index Primer Pairs (New England Biolabs, cat# E6442S), with 15 cycles of PCR performed.

All amplified libraries were quantified by Qubit dsDNA HS Kit and run on the Agilent 4200 Tapestation System (Agilent, cat# G2991AA) using the High Sensitivity D5000 Screentape (Agilent, cat# 5067-5592) and High Sensitivity D5000 Reagents (Agilent, cat# 5067-5593).

Libraries were sequenced on Illumina's NextSeq 550 System (Illumina, cat# SY-415-1002) generating 2-12 million paired-end reads per sample for clinical isolates and approximately 30 million reads for rescued viruses.

CVR-GLA-3 was re-sequenced using amplicon sequencing, to confirm consensus. Briefly, the sequencing library was prepared according to the ARTIC nCoV-2019 sequencing protocol version 2 https://artic.network/ncov-2019 until the amplicon generation step. Generated amplicons were used to prepare Illumina sequencing libraries using Kapa LTP Library as described above. Sequencing of libraries was carried out on Illumina's MiSeq system (Illumina, cat# SY-410-1003)

using a MiSeq Reagent v2 500 cycle nano kit (Illumina, cat# MS-103-1003). Sequencing generated approximately 0.3 million paired end reads per sample.

Passaging the wt reverse genetics (RG) plasmid. The wt pCC1-4K-SARS-CoV-2 plasmid was transformed by electroporation (BioRad Gene Pulser Xcel) using TransforMax™ EPI300™ Electrocompetent *E. coli*. This was considered P0. Two colonies were picked (to create 2 plasmid lineages) and grown for 6 hours in identical 2 ml cultures of Tryptic Soy Broth (Becton Dickinson) supplemented with chloramphenicol at 12.5 μg/ml. The cultures were diluted 1:100 and plated on fresh plates (considered P1). This process was repeated until the P5 plate (5x 6 hr liquid cultures and 6x solid cultures). Plasmid DNA from one colony from each P5 plate was generated as follows. A 10 hr 2 ml culture was diluted to a 12 hr 200 ml culture, which was diluted 1:5 with growth media and induced by adding D-arabinose to a final concentration 0.1%. This culture was incubated at 37°C for 5 hrs before bacteria were lysed and endotoxin free DNA was extracted using Nucleobond Xtra Midi EF kit (Machery Nagel, 740420.50). The conservative estimate of >100 doublings is based upon 1 doubling per hour in 5x 6 hr liquid cultures (30) and 6x12 hr solid phase cultures (72) and does not take into account the doublings required to make the 1L culture for plasmid extraction.

SARS-CoV-2 virus passage. Cells were seeded the day before infection in T25 flasks ($7x10^5$ cells per flask for GLA1 passage series) or in 6-well plates ($4x10^5$ cells per well for mCherry passage series) in DMEM supplemented with 10% FCS before being infected using 0.01 pfu/cell (GLA-1) or 0.1 (mCherry) pfu/cell. The passage series

indicated in the figures was followed. Immediately before each passage, medium was removed from the fresh target cells and replaced with DMEM supplemented with reduced FCS (2-4%). At each passage, virus-containing supernatant was harvested and clarified by low-speed spin. The fresh cells were inoculated with virus-containing supernatant diluted ~1:100 (Vero E6) or 1:200 (AAT, the increased dilution factor taking into account the increased susceptibility of these cells to SARS-CoV-2 infection). At the indicated points, 250 μl of virus-containing supernatant was mixed with TRIzol LS (Thermo Fisher) and RNA was extracted using a hybrid Trizol-RNeasy protocol.

SARS-CoV-2 fluorescent plaque assays. Plaque assays of passages 3-5 were conducted as described in Plaque Assay section above. Fixed cells were scanned on the Celigo Imaging Cytometer (Nexcelom Bioscience) using the red channel to visualize mCherry or green channel to visualize ZsGreen. The cells were then subsequently stained with Coomassie staining solution (2 % Coomassie Brilliant Blue R-250, 50 % ethanol, 7.5% acetic acid) for 30 min then rinsed with water. The Coomassie stained cells were imaged on the brightfield channel of the Celigo Imaging Cytometer. Because the 6-well format is relatively large, the presented images are composites of multiple Celigo images taken of the same wells. The stability of the mCherry reporter virus was assessed by scoring the percentage of plaques visible following Coomassie staining that were mCherry-positive in the linear range of the dilution series (total plaque number in the range of 22-55 for each replicate).

Quantification of mCherry and N-expressing cells. To quantify the proportion of cells positive for mCherry and for N protein, Vero E6 cells seeded in 24-well plates were infected with passage 3 SARS-CoV-2-mCherry at an MOI of 0.1 for 48 h. Fixed and permeabilised cells were then stained for N protein using the primary sheep anti-N (DA114, 5th bleed) antibody at 1:500, followed by a secondary mouse anti-sheep IgG-FITC labelled antibody (Sigma, F4891) used at 1:100, and with Hoechst 33342 (2 μg/ml) as described above. Cells were imaged using the Celigo Imaging Cytometer and Hoechst-positive cells were gated into populations based on mean fluorescent intensity of the green and red channels to identify the proportion of infected cells positive for mCherry and/or N protein. Virus infections were performed in triplicate and at least 45000 cells were quantified for each sample.

Sequencing of reverse genetics (RG) plasmids. Purified plasmid DNA was sheared to ~300bp using a Covaris S220 Focused-ultrasonicator (Covaris, cat# 500217). Approximately 40ng of sheared DNA was used to prepare sequencing libraries using Kapa HyperPrep kit (Kapa Biosystems, cat# KK8504). The samples were uniquely indexed using the NEBNext Multiplex Oligos for Illumina 96 Unique Dual Index Primer Pairs Set 2 (New England Biolabs, cat# E6442S) and 5 cycles of PCR were performed. The amplified libraries were quantified by Qubit dsDNA HS Kit and run on the Agilent 4200 Tapestation System (Agilent, cat# G2991AA) using the High Sensitivity D5000 Screentape (Agilent, cat# 5067-5592) and High Sensitivity D5000 Reagents (Agilent, cat# 5067-5593). The sequencing of the libraries was carried out on Illumina's MiSeq system (Illumina, cat# SY-410-1003) using a MiSeq Reagent v2 500 cycle nano kit

(Illumina, cat# MS-103-1003). Sequencing generated approximately 0.3 million paired-end reads per sample.

Bioinformatic analyses. For CVR-GLA-1 and CVR-GLA-3, reads were quality-trimmed using TrimGalore, aligned to the SARS-CoV-2 Wuhan-Hu-1 reference sequence (accession MN908947) using BWA (Li and Durbin, 2009), and the consensus was called on all sites with a depth greater than 10 reads using iVar (Grubaugh *et al.*, 2019); for the CVR-GLA-3 (CVR3899) sample re-sequenced using amplicons, iVar was also used to trim ARTIC primers from the aligned reads. Mutations with frequencies above 10% were extracted from the read alignment using DiversiTools (http://josephhughes.github.io/DiversiTools/). Rescued viruses were first *de novo* assembled using SPAdes (Bankevich *et al.*, 2012) to identify the sequence and location of the reporter gene (mCherry, ZsGreen or NLuc), based on the Wuhan-Hu-1 reference sequence, leading to the manual creation of a new reference sequence for each rescued virus. Samples were subsequently aligned to their corresponding reference sequence and checked for any consensus level mutations or indels using BWA and iVar as described previously; no consensus level mutations or indels were observed. For plasmids, a reference sequence for the mCherry reporter containing plasmid was already available (pCC1-4K-SARS-CoV-2-mCherry), this was manually modified to create a corresponding sequence for the wild type, ZsGreen and NLuc reporter-containing plasmid, using the obtained *de novo* sequences from the rescued viruses. Samples were subsequently aligned to their corresponding reference sequence and checked for any consensus level mutations or indels using BWA and iVar as described previously; no consensus level mutations or indels were observed.

For CVR-GLA-2, mapping of the SARS-CoV-2-specific reads was performed using the in-house, reference-based assembly tool Tanoti (http://www.bioinformatics.cvr.ac.uk/tanoti.php), with Wuhan-Hu-1 (NC_045512.2) as the reference sequence. A consensus sequence was produced from the resulting SAM file. Analysis of the sequence was performed using the online bioinformatic tool CoV-GLUE (http://cov-glue.cvr.gla.ac.uk/). To investigate the low frequency diversity present in plasmid and rescued virus samples, mutation frequencies were calculated at all genome positions that had a minimum coverage of 100 and using a minimum quality score of 30 using DiversiTools; a conservative arbitrary frequency threshold of 1% was selected to distinguish substitutions from sequencing errors and artefacts. Sequence reads for the pCC1-4K plasmids and rescued viruses, and clinical isolates, can be accessed in the GenBank BioProject PRJNA658321.

Metagenomics results. To determine whether contamination was present in the clinical isolates, the quality-filtered reads for CVR-GLA-1 and CVR-GLA-3 were compared to the NCBI RefSeq database (O'Leary *et al.*, 2016) using DIAMOND blastx (Buchfink, Xie and Huson, 2015). For the CVR-GLA-3 clinical isolate 1,909,732 reads were produced and 28% of all DIAMOND blastx hits were to viruses, and all but one of these was to the SARS-CoV-2 containing Sarbecovirus genus; a single read gave a distant hit to Brochothrix phage A9. The remaining reads were predominantly classified as Eukaryotic (56%; 39% to the subphylum Vertebrata), with 11% of reads hitting to bacteria (predominantly *Acinetobacter baumannii*, *Staphylococcus agnetis* and *Clostridioides difficile*). 12,005,902 reads were obtained in CVR-GLA-1 grown in

Vero E6 cells, 85% of the 9,007,664 reads with matches to refseq matched SARS-CoV-2 with most other reads matching *Homo sapiens* followed by *Plasmodium ovale wallikeri*. A small number of reads matched fungi and bacteria indicating a clean cell and virus culture.

Plaque assays. Monolayers of A549, Vero E6 cells, and their ACE2/TMPRSS2 derivatives were prepared by seeding 2.5 x 10^5 cells/well in 12-well plates and incubating the cells overnight at 37°C and 5% CO_2. Serial dilutions of virus were prepared in DMEM/2% FCS/1 x NEAA, and 150 μl was used to infect cell monolayers following the aspiration of media. The infection was incubated for 1 h at 37°C and 5% CO_2 with occasional tilting to distribute the inoculum evenly. A 1 ml overlay of MEM/0.6% Avicel RC-591/2 % FCS was added to each well and cells were incubated undisturbed for 72 hrs at 37°C and 5% CO_2. The cells were fixed by submerging in PBS/8% formaldehyde for a minimum of 1 hr. Cells were washed twice with PBS before staining with Coomassie Blue staining solution (0.1 % Coomassie Brilliant Blue R-250/45% methanol/10% acetic acid) for 30 minutes to visualize plaques, and were finally washed with water. Images of plaque assays were acquired using a photo scanner (Epson Expression 1680 Pro). Image brightness was adjusted in ImageJ and photomicrographs were cropped and posterized in Microsoft PowerPoint.

To quantify plaque area, the brightness and contrast of scan images were adjusted, the image desaturated and then posterized to 2 levels in GIMP-2.10. Plaque area was quantified in ImageJ-1.53a (Schneider, Rasband and Eliceiri, 2012) by using the wand tracing tool to trace around the edge of isolated plaques and using the measure command. To calculate plaque area in mm^2, the wand tracing tool was used

to quantify the mean pixel area of three independent uninfected monolayers which were assumed to have an area of 383.6 mm^2, in accordance with dimensions provided by the plate supplier (Corning 12-well plates, cat# 3513).

Well-clearance/monolayer integrity CPE assay. Vero E6-ACE2 (VA), Vero E6-ACE2-TMPRSS2 (VAT), A549-ACE2 (AA) and A549-ACE2-TMPRSS2 (AAT) cells were seeded in optical 96-well plates (Perkin Elmer CellCarrier-96 Ultra Microplates Cat# 6055302) at 0.14x10^5 cells/well and incubated overnight. The following day, the cells were incubated with a panel of 2-fold serially diluted compounds from MedChemExpress (10-point curves in columns 1-10) where the concentrations ranged from 10 μM to 20 nM. The compounds were dissolved in DMSO and columns 11 and 12 were incubated with the equivalent dose of DMSO to the highest dose of DMSO used in the 10 μM incubations. The cells were then mock-infected or infected with a predetermined dose of SARS-CoV-2 that would cause substantial CPE by 72 hrs post-infection. At 72 hrs post-infection, cells were fixed with 8% formaldehyde and stained with Coomassie staining solution (0.1% Coomassie Brilliant Blue R-250/45% methanol/10% acetic acid). The air-dried plates were scanned using a Celigo imaging cytometer (Nexcelom), allowing monolayer integrity to be quantified. Quantification of monolayer integrity in the presence of virus was used to assess virus replication and was used to assess compound toxicity in the absence of virus. Assays were 'multiplexed' by executing dead cell protease assays in the same plates (supernatant transferred to a separate assay plate) using the CytoTox-Glo™ Cytotoxicity Assay (Promega) in accordance with the manufacturer's instructions and the resulting bioluminescence was measured using a GloMax Luminometer (Promega). For the

dose response curves, virus replication was calculated by comparing the well-clearance to infected DMSO controls (100% replication) and uninfected DMSO controls (0% replication) on the same plate. Similarly, toxicity was normalised to infected DMSO controls (100% toxicity) and uninfected DMSO controls (0% toxicity). The curves represent the mean and standard error from 4 replicate experiments (duplicate plates in two independent experiments). EC_{50} values were interpolated using GraphPad Prism. Normalised toxicity values above 100% or below 0% were assigned 100% or 0% values respectively.

Generation of anti-coronavirus polyclonal antibodies. For the sheep polyclonal antibodies generated for this study, individual SARS-CoV-2, SARS-CoV, MERS-CoV, HCoV 229E or HCoV OC43 ORFs were cloned into pGex (carrying a GST tag) and pMex (MBP tag), in order to yield GST- and MBP-tagged coronavirus proteins. The N-terminal GST fusion proteins for each coronavirus protein were used as antigens, and sheep were immunised with each antigen, followed by up to 5 further injections 28 days apart, with bleeds performed 7 days after each injection. Antibodies were subsequently affinity purified from serum using N-terminal MBP-tagged recombinant protein. The SARS-CoV-2 S, S-RBD and ORF7a antibodies were generated slightly differently, as for these N-terminal MBP tagged proteins were used as antigens, and upon harvest, serum antibodies were affinity-purified using the MBP-tagged protein, followed by depletion of anti-MBP antibodies (to remove the antibodies present in the serum against MBP). Total IgGs from the serum containing antibodies to the full-length S of SARS-CoV-2 were purified using a protein G sepharose, as not enough protein was available to generate an affinity column. Further details (including

relevant accession numbers) of the specific protein sequences used as antigens for each protein can be found in S2 Table and at the website: https://mrcppu-covid.bio/.

Immunofluorescence for antibody validation. For the IF used in the validation of the coronavirus antibodies generated for this study, 2.5 x 10^4 Vero E6 cells were seeded on glass coverslips in 24 well plates and either uninfected (mock) or infected with SARS-CoV-2 England-02 (EPI_ISL_407073) at a MOI of 0.1. At 48 hrs post infection, cells were fixed and permeabilised in 8% formaldehyde/1% Triton X-100. DAPI (Thermo Fisher Scientific, cat# P36395) was included in the mounting medium, and slides were subsequently incubated with the primary sheep anti-coronavirus antibodies at 1:500 dilution, followed by incubation with a rabbit anti-sheep Alexa Fluor 555 secondary antibody (Abcam cat# ab150182) at a 1:1000 dilution. Cells were imaged with a Zeiss LSM 880 confocal, with 1 Airy unit pinhole for all channel acquisitions, and scanned with a 0.07 μm x 0.07 μm pixel size. The objective lens used was a Plan-Apochromat 63x/1,4NA (M27). Alexa Fluor 555 was excited using a 514nm laser line and detected in the 547-697nm range. DAPI was excited using a 405nm laser line and detected in the 410-559nm range. Post-acquisition, matched pairs of mock and infected images for each antibody were optimised to the same degree for contrast using Zeiss Zen software. Images were acquired as 12-bit *.czi files and exported as 8-bit TIFF files. For investigations of IF of the newly reported clinical isolates, the conditions were the same, with the exception that A549-ACE2-TMPRSS2 cells were either uninfected (mock) or infected with CVR-GLA-1, CVR-GLA-2, CVR-GLA-3 or England-02 at a MOI of 0.01 for 48 hrs prior to fixation/permeabilisation. All other methods were as described above.

Immunofluorescence of infected cells with rescued SARS-CoV-2. To titre the rescued P0 virus stock, Vero E6 cells were seeded in 12-well plates. The following day, these cells were infected with 10-fold serially diluted inocula in 2% DMEM (at this point the cells had reached 100% confluency and were infected using 200μl of inoculum per well). Following incubation for 1hr, the cells were covered with overlay media composed of equal volumes of 2XMEM (Thermo Fisher Scientific) supplemented with 4% FCS and 1.2% Avicel in RO H_2O. The virus propagation was allowed to continue for 48 hrs. The cells were then fixed using 8% formaldehyde for 1 hr followed by immunostaining. The plates were washed twice with PBS and then permeabilized using 0.5% Triton X-100 in PBS for 5 minutes. The plates were then washed thrice with PBS. Blocking of the wells was carried out for 2 hrs using PBS supplemented with 5% FCS (5% FCS in PBS). Infected cells were probed overnight using anti-N protein (SARS-CoV-2) using the sheep-derived antibody described herein (Dundee, 3rd bleed, 0.39mg/ml), used at a 1:500 dilution in 5% FCS in PBS. The plates were washed thrice with PBS. To detect the primary antibody, the cells were incubated with Alexa Fluor 488 conjugated donkey anti-sheep antibody (A11015, Thermo Fisher Scientific) diluted 1:1000 in 5% FCS in PBS for 1 hr. The plates were washed thrice with PBS and scanned using a Celigo imaging cytometer (Nexcelom) at 2 μm resolution.

Immunofluorescence quantification of ACE2. Cells were seeded on glass coverslips (VWR) in 24-well plates at a confluency of 6 x 10^4 cells/well. The following day, cells were washed with PBS prior to fixation and a subsequent 3 washes with

PBS. Cells were blocked for 30 min with PBS/10% mouse serum (Abcam)/1% BSA fraction V (Sigma). The goat polyclonal anti-ACE2 antibody (R&D Systems, AF933) was used at a dilution of 1:100 in the blocking solution described above overnight at 4°C. The cells were then washed three times with PBS and probed with a mouse anti-goat IgG FITC-conjugated secondary antibody (Sigma, F4891) used at 1:100 and stained with Hoechst 33342 (2 μg/ml) at room temperature for 30 min before washing 4 times with PBS. Finally, actin was stained using Alexa Fluor™ 568 Phalloidin (Invitrogen) at 1:50 in PBS for 20 minutes before washing 4 times with PBS. Coverslips were then mounted on glass microscopy slides (Thermo Scientific) with ProLong™ Gold Antifade mountant (ThermoFisher Scientific).

Images of cell monolayers were acquired with equal gain, laser power, pinhole and scaling of 0.008 μm pixel size in x and y using a Zeiss LSM 880 confocal microscope. The ACE2-FITC was excited at 488nm and detected in the 493-566nm range and Hoechst 33342 was excited at 405nm and detected in the 410-489nm range. Post-acquisition, images within each set were optimised to the same degree for contrast using Zen software (Carl Zeiss). Images were acquired as 12-bit *.czi files and exported as 8-bit TIFF files. The objective lens used was a Plan-Apochromat 40x/1.4NA M27 (Carl Zeiss). To quantify ACE2 levels, 12-bit images of cell monolayers were acquired with equal gain, laser power, pinhole and scaling of 0.008 μm pixel size in x and y using a Zeiss LSM 880 confocal microscope. The objective lens used was a Zeiss Plan-Apochromat 40x/1.4NA M27. The ACE2-FITC was excited at 488nm and detected in the 493-566nm range and Hoechst was excited at 405nm and detected in the 410-489nm range. To define whole cells, actin was stained with

phalloidin-Alexa 568 which was excited at 561nm and detected at 568-712nm. ACE2 intensity values were measured using Cell Profiler (cellprofiler.org).

Rescue of infectious SARS-CoV-2 from plasmid. The rescue process is facile and has been independently executed multiple times in the authors' laboratories in Estonia, the UK and Australia using different protocols.

Rescue protocol 1: Ten micrograms (*µ*g) of full-length RG SARS-CoV-2 (Wuhan-Hu-1 strain) infectious clone cDNA was transfected into BHK-21 using Lipofectamine LTX (Life Technologies) according to the manufacturer's instructions. Transfected BHK-21 cells were cultured in Opti-MEM medium supplemented with 2% FCS and incubated at 37° C with 5% CO_2 for 48-72 hrs or until 50% cytopathic effect (CPE) was observed by light microscopy. The supernatant was subsequently collected and transferred to Vero E6 cells, which were cultured in DMEM medium supplemented with 2% FCS. Supernatant was collected after 48-72 hrs, or until 50% CPE was observed, then centrifuged to exclude cell debris, filtered through a 0.22 *µ*m filter, aliquoted and stored at -80ºC prior to titration.

Rescue protocol 2: BHK-21 cells were grown in 6-well plates in DMEM supplemented with 10% FCS. When cells reached 80% confluency the media was changed to DMEM supplemented with 2% FCS. BHK-21 cells were transfected with Lipofectamine LTX with PLUS reagent (Thermo Fisher Scientific) using the following mix: 9 *µ*l of Lipofectamine LTX, 3 *µ*g of plasmid DNA (containing virus cDNA) and 3 *µ*l of PLUS reagent were added to 150 *µ*l of OptiMEM (Thermo Fisher Scientific). After mixing, the complexes were allowed to form for 20 minutes before pipetting the mixture onto the BHK-21 cells. On

the following day, the media on Vero E6 cells in T25 flasks was changed to 3 ml of 2% DMEM. 1 ml of media from the transfected BHK-21 cells was then added to the Vero E6 culture and 1ml of fresh 2% DMEM was added to the BHK-21 cells. On the next day, the supernatant transfer process was repeated. Inoculation using supernatant from BHK-21 cells was repeated a final time on the third day post-transfection. Virus was allowed to replicate in Vero E6 cells for a further 4 days before harvesting the P0 stock.

Immunoprecipitation. To prepare lysates for IP, Vero E6 cells were infected with SARS-CoV2 (England-02 2020 EPI_ISL_407073) at a MOI of 0.1 for 3 days or were left uninfected. At 3 days post infection, cells were trypsinised and rinsed in ice-cold PBS before being lysed in ice cold lysis buffer (50 mM Tris/HCl pH 7.5, 1 mM EGTA, 1 mM EDTA, 1% (v/v) Triton X-100, 1 mM sodium ortho-vanadate, 50 mM NaF, 5 mM sodium pyrophosphate, 0.27 M sucrose, 10 mM sodium 2-glycerophosphate, 1 mM phenylmethylsulphonyl fluoride, 1 mM benzamidine) (Emmerich *et al.*, 2016). Protein concentrations were measured using the Bradford method and IPs were set up overnight at 4°C with the antibody and protein lysate in a ratio of 3 μg of antibody per mg of protein lysate. After overnight incubation at 4°C, IPs were washed thrice in the same lysis buffer as mentioned above, before being denatured with 1X LDS (Thermo Fisher Scientific, NP0008) and 1X sample reducing agent (Thermo Fisher Scientific, NP0009). The IPs were subjected to SDS-PAGE and then transferred onto Immobilon-FL PVDF membranes (Merck Millipore, IPFL00010) before being blocked for 1 hr in 5% skimmed milk in 1X Tris-buffered saline containing 0.2% Tween 20 (TBST). The PVDF membranes were then incubated with their respective biotinylated primary

antibodies (0.5 – 1.0 mg/ml) overnight at 4°C. The biotinylation was performed using a kit (Thermo Fisher Scientific, 90407). Streptavidin Protein DyLight 680 (Thermo Fisher Scientific, 21848) or Streptavidin Protein DyLight 800 (Thermo Fisher Scientific, 21851) were used instead of a secondary at a concentration of 1:5000-1:10000. The blots were scanned using the LI-COR Odyssey CLx imaging system.

Western blotting and luciferase assay. For validation of the antibodies generated for this resource that were raised against various coronaviral proteins, Vero E6 cells were either mock-infected or infected with SARS-CoV-2 England-02 (hCoV-19/England/02/2020, GISAID accession EPI_ISL_407073) at an MOI of 0.1 or 1 (as specified) for 72 hrs before being harvested (in protein sample buffer) and sonicated. For confirmation of viral protein expression from the rescued viruses, Vero E6 cells were uninfected (mock) or infected with RG-rescued SARS-CoV-2-Wuhan-Hu-1 (wt) alongside mCherry, ZsGreen and Nanoluciferase derivatives for 48 hrs before being harvested (in protein sample buffer) and sonicated. Lysed cells were processed using a Nano-Glo Luciferase Assay kit (Promega, Cat# N1130) and bioluminescence measured using a GloMax Luminometer (Promega).

For western blots the cell lysates were resolved using Novex 4-12% acrylamide gels (Life Technologies) before wet-transfer onto nitrocellulose membranes (Sigma Aldrich, cat# GE10600003), after which, membranes were probed with the indicated primary antibody diluted to 0.25 μg/ml (for the coronavirus antibodies generated for this study) in SEA BLOCK (Thermo Scientific, cat# 37527), unless otherwise specified. The primary antibody was subsequently visualized by probing with a fluorescently labeled (DyLight) rabbit anti-sheep IgG secondary antibody (Rabbit anti-sheep IgG

(H+L) Cross-Adsorbed Secondary Antibody Dylight 800, Thermo Fisher Scientific, cat# SA5-10060) diluted 1:10000 prior to scanning with a Li-Cor Odyssey scanner.

To visualize the endogenous and exogenous expression of ACE2 and TMPRSS2, Vero E6 and A549 cells that were either not modified or transduced with lentiviral vectors encoding TMPRSS2 and/or ACE2 (LV-Hygro-ACE2 or LV-Neo-TMPRSS2) before being lysed and treated as above. Membranes were then probed with either anti-ACE2 (R&D Systems cat# AF933) or anti-TMPRSS2 (Proteintech cat#14437-1-AP or Abcam, cat# ab92323) primary antibodies before visualisation with fluorescently labeled (DyLight) donkey anti-goat IgG and goat anti-rabbit IgG secondary antibodies (Thermo Fisher Scientific, cat# SA5-10090 and SA5-10036) diluted 1:10000 and scanned with a Li-Cor Odyssey scanner.

For loading controls, actin was visualised using an anti-actin antibody (JLA20 hybridoma, provided by the Developmental Studies Hybridoma Bank at the University of Iowa), in conjunction with a fluorescently labeled (DyLight) goat anti-mouse secondary antibody (Thermo Fisher Scientific, cat# SA5-10176).
