# Supplementary material for: A plasmid DNA-launched SARS-CoV-2 reverse genetics system and coronavirus toolkit for COVID-19 research
Source: PLoS Biol. 2021 Feb 25;19(2):e3001091. doi: 10.1371/journal.pbio.3001091 (PMC7906417; doi:10.1371/journal.pbio.3001091)
Supplement: S2 Table — (PDF) [file pbio.3001091.s009.pdf]

S2 Table. Reagents and Resources

REAGENT or RESOURCE	SOURCE	IDENTIFIER
**Antibodies**		
Goat polyclonal anti-ACE2	R&D Systems	Cat#AF933; RRID: AB_355722
Rabbit polyclonal anti-TMPRSS2	Proteintech	Cat#14437-1-AP
Mouse monoclonal anti-SARS-CoV/SARS-CoV-2 Nucleocapsid (N)	SinoBiologicals	40143-MM08
Rabbit monoclonal [EPR3861] to TMPRSS2	Abcam	Abcam Cat# ab92323, RRID:AB_10585592
Mouse monoclonal anti-Actin	Developmental Studies Hybridoma Bank, University of Iowa	Clone JLA20
Rabbit anti-sheep IgG (H+L) Cross Adsorbed Secondary Antibody, DyLight 800	Thermo Fisher Scientific	Cat# SA5-10060
Goat anti-Mouse IgG (H+L) Cross-Adsorbed Secondary Antibody, DyLight 800	Thermo Fisher Scientific	Cat# SA5-10176
Goat anti-Rabbit IgG (H+L) Cross-Adsorbed Secondary Antibody, DyLight 800	Thermo Fisher Scientific	Cat# SA5-10036
Donkey anti-Goat IgG (H+L) Cross-Adsorbed Secondary Antibody, DyLight 680	Thermo Fisher Scientific	Cat# SA5-10090
Streptavidin Protein DyLight 680	Thermo Fisher Scientific	Cat# 21848
Streptavidin Protein DyLight 800	Thermo Fisher Scientific	Cat# 21851
Rabbit anti-sheep IgG H&L Alexa Fluor 555	Abcam	Cat# ab150182
Goat anti-mouse IgG H+L Alexa Fluor 488	Life Technologies	Cat#A28175
Mouse monoclonal anti-Goat/Sheep IgG−FITC	Sigma	Cat#F4891
SARS-CoV-2 orf6	This paper; https://mrcppu-covid.bio/	Cat# DA087
SARS-CoV-2 orf8	This paper; https://mrcppu-covid.bio/	Cat# DA088
SARS-CoV-2 orf9a	This paper; https://mrcppu-covid.bio/	Cat# DA089
SARS-CoV-2 orf10	This paper; https://mrcppu-covid.bio/	Cat# DA090
SARS-CoV-2 nsp10	This paper; https://mrcppu-covid.bio/	Cat# DA091
SARS-CoV-2 orf7b	This paper; https://mrcppu-covid.bio/	Cat# DA092
SARS-CoV-2 nsp7	This paper; https://mrcppu-covid.bio/	Cat# DA093
SARS-CoV-2 nsp9	This paper; https://mrcppu-covid.bio/	Cat# DA094
SARS-CoV-2 orf3b	This paper; https://mrcppu-covid.bio/	Cat# DA100
SARS-CoV-2 orf9b	This paper; https://mrcppu-covid.bio/	Cat# DA101
SARS-CoV-2 orf3a	This paper; https://mrcppu-covid.bio/	Cat# DA102
SARS-CoV-2 nsp1	This paper; https://mrcppu-covid.bio/	Cat# DA103
SARS-CoV-2 nsp2	This paper; https://mrcppu-covid.bio/	Cat# DA105
SARS-CoV-2 M	This paper; https://mrcppu-covid.bio/	Cat# DA107

SARS-CoV-2 E	This paper; https://mrcppu-covid.bio/	Cat# DA108
SARS-CoV-2 nsp8	This paper; https://mrcppu-covid.bio/	Cat# DA110
SARS-CoV-2 nsp13	This paper; https://mrcppu-covid.bio/	Cat# DA111
SARS-CoV-2 nsp14	This paper; https://mrcppu-covid.bio/	Cat# DA112
SARS-CoV-2 nsp16	This paper; https://mrcppu-covid.bio/	Cat# DA113
SARS-CoV-2 N	This paper; https://mrcppu-covid.bio/	Cat# DA114
SARS-CoV-2 nsp5	This paper; https://mrcppu-covid.bio/	Cat# DA118
SARS-CoV-2 nsp11/12	This paper; https://mrcppu-covid.bio/	Cat# DA119
SARS-CoV-2 nsp15	This paper; https://mrcppu-covid.bio/	Cat# DA120
SARS-CoV-2 S	This paper; https://mrcppu-covid.bio/	Cat# DA123
SARS-CoV-2 orf7a	This paper; https://mrcppu-covid.bio/	Cat# DA124
SARS-CoV-2 S-RBD	This paper; https://mrcppu-covid.bio/	Cat# DA125
SARS-CoV-2 nsp3	This paper; https://mrcppu-covid.bio/	Cat# DA126
SARS-CoV N	This paper; https://mrcppu-covid.bio/	Cat# DA106
MERS-CoV N	This paper; https://mrcppu-covid.bio/	Cat# DA117
229e N	This paper; https://mrcppu-covid.bio/	Cat# DA115
OC43 N	This paper; https://mrcppu-covid.bio/	Cat# DA116
SARS-CoV E	This paper; https://mrcppu-covid.bio/	Cat# DA096
MERS-CoV E	This paper; https://mrcppu-covid.bio/	Cat# DA095
**Bacterial and Virus Strains**		
CVR-GLA-1	This paper	Genbank accession: MT882022
CVR-GLA-2	This paper	GenBank accession: MT906650
CVR-GLA-3	This paper	GenBank accession: MT906649
hCoV-19/England/02/2020 (England-02)	Public Health England	GISAID accession: EPI_ISL_407073
TransforMax™ EPI300™ Electrocompetent E. coli	Cambio	EC300110
**Clinical Samples**		
CVR837 sputum (for isolation of CVR-GLA-1 SARS-CoV-2 virus)	NHS Greater Glasgow & Clyde	GISAID accession: EPI_ISL_461705
CVR2224 sputum (for isolation of CVR-GLA-2 SARS-CoV-2 virus)	NHS Greater Glasgow & Clyde	GISAID accession: EPI_ISL_448167
CVR3899, bronchoalveolar lavage (BAL) (for isolation of CVR-GLA-3 SARS-CoV-2 virus)	NHS Greater Glasgow & Clyde	GISAID accession: EPI_ISL_490695

**Recombinant Proteins and Chemicals**		
SARS-CoV-2 orf6 protein, N-term GST uncleaved	This paper; https://mrcppu-covid.bio/	Cat# DU68490, accession: QHD43420.1
SARS-CoV-2 orf8 protein, N-term GST uncleaved	This paper; https://mrcppu-covid.bio/	Cat# DU68491, accession: QHD43422.1
SARS-CoV-2 orf9a protein, N-term GST uncleaved	This paper; https://mrcppu-covid.bio/	Cat# DU68492, accession: MN908947.3
SARS-CoV-2 orf10 protein, N-term GST uncleaved	This paper; https://mrcppu-covid.bio/	Cat# DU67614, accession: QHI42199.1
SARS-CoV-2 nsp10 protein, N-term GST uncleaved	This paper; https://mrcppu-covid.bio/	Cat# DU68489, accession: QHD43415.1
SARS-CoV-2 orf7b protein, N-term GST uncleaved	This paper; https://mrcppu-covid.bio/	Cat# DU67625, accession: QJC19833.1
SARS-CoV-2 nsp7 protein, N-term GST uncleaved	This paper; https://mrcppu-covid.bio/	Cat# DU68505, accession: QHD43415.1
SARS-CoV-2 nsp9 protein, N-term GST uncleaved	This paper; https://mrcppu-covid.bio/	Cat# DU68506, accession: QHD43415.1
SARS-CoV-2 orf3b protein, N-term GST uncleaved	This paper; https://mrcppu-covid.bio/	Cat# DU68507, accession: QHD43419.1
SARS-CoV-2 orf9b protein, N-term GST uncleaved	This paper; https://mrcppu-covid.bio/	Cat# DU68493, accession: MN908947.3
SARS-CoV-2 orf3a protein, N-term GST uncleaved	This paper; https://mrcppu-covid.bio/	Cat# DU67698, accession: QHD43417.1
SARS-CoV-2 nsp1 protein, N-term GST uncleaved	This paper; https://mrcppu-covid.bio/	Cat# DU66413, accession: QHD43415.1
SARS-CoV-2 nsp2 protein, N-term GST uncleaved	This paper; https://mrcppu-covid.bio/	Cat# DU66414, accession: QHD43415.1
SARS-CoV-2 M protein, N-term GST uncleaved	This paper; https://mrcppu-covid.bio/	Cat# DU67699, accession: QHD43419.1
SARS-CoV-2 E protein, N-term GST uncleaved	This paper; https://mrcppu-covid.bio/	Cat# DU68523, accession: QHD43418.1
SARS-CoV-2 nsp8 protein, N-term GST uncleaved	This paper; https://mrcppu-covid.bio/	Cat# DU66416, accession: QHD43415.1
SARS-CoV-2 nsp13 protein, N-term GST uncleaved	This paper; https://mrcppu-covid.bio/	Cat# DU66417, accession: QHD43415.1
SARS-CoV-2 nsp14 protein, N-term GST uncleaved	This paper; https://mrcppu-covid.bio/	Cat# DU66418, accession: QHD43415.1
SARS-CoV-2 nsp16 protein, N-term GST uncleaved	This paper; https://mrcppu-covid.bio/	Cat# DU66420, accession: QHD43415.1

SARS-CoV-2 N protein, N-term GST uncleaved	This paper; https://mrcppu-covid.bio/	Cat# DU67726, accession: QHD43423.2
SARS-CoV-2 nsp5 protein, N-term GST uncleaved	This paper; https://mrcppu-covid.bio/	Cat# DU67779, accession: QHD43415.1
SARS-CoV-2 nsp11/12 protein, N-term GST uncleaved	This paper; https://mrcppu-covid.bio/	Cat# DU67736, accession: QHD43415.1
SARS-CoV-2 nsp15 protein, N-term GST uncleaved	This paper; https://mrcppu-covid.bio/	Cat# DU66419. accession: QHD43415.1
SARS-CoV-2 S protein, N-term MBP uncleaved	This paper; https://mrcppu-covid.bio/	Cat# DU67743, accession: QHD43416.1
SARS-CoV-2 orf7a protein, N-term MBP uncleaved	This paper; https://mrcppu-covid.bio/	Cat# DU68508, accession: QHD43421.1
SARS-CoV-2 S-RBD protein, N-term MBP uncleaved	This paper; https://mrcppu-covid.bio/	Cat# DU67753, accession: QHD43416.1
SARS-CoV-2 nsp3 protein, N-term GST uncleaved	This paper; https://mrcppu-covid.bio/	Cat# DU67768, accession: QHD43415.1
SARS-CoV N protein, N-term GST uncleaved	This paper; https://mrcppu-covid.bio/	Cat# DU67701, accession: NP_828858.1
MERS-CoV N protein, N-term GST uncleaved	This paper; https://mrcppu-covid.bio/	Cat# DU67732, accession: ANF29169.1
229e N protein, N-term GST uncleaved	This paper; https://mrcppu-covid.bio/	Cat# DU67703, accession: NP_073556.1
OC43 N protein, N-term GST uncleaved	This paper; https://mrcppu-covid.bio/	Cat# DU67704, accession: AEN19370.1
SARS-CoV E protein, N-term GST uncleaved	This paper; https://mrcppu-covid.bio/	Cat# DU68502, accession: NP_828854.1
MERS-CoV E protein, N-term GST uncleaved	This paper; https://mrcppu-covid.bio/	Cat# DU68503, accession: AGV08472.1
Nafamostat mesylate	MedChemExpress	Cat# HY-B0190A
Apilimod	MedChemExpress	Cat# HY-14644
EIDD_2801	MedChemExpress	Cat# HY-135853
Remdesivir	MedChemExpress	Cat# HY-104077
ProLong Gold Antifade Mountant with DAPI	Thermo Fisher Scientific	Cat# P369235
**Commercial Assays**		
Pierce Antibody Biotinylation Kit for IP	Thermo Fisher Scientific	Cat# 90407
Agencourt RNA Clean AMPure XP Beads	Beckman Coulter	Cat# A63987
Qubit dsDNA HS Kit	Thermo Fisher Scientific	Cat# Q32854
Kapa LTP Library Preparation Kit for Illumina Platforms	Kapa Biosystems	Cat# KK8232
Illumina Nextera DNA Flex Kit	Illumina	Cat# 20018704
Illumina Nextera DNA Unique Dual Indexes Set A	Illumina	Cat# 20027213
Agilent 4200 Tapestation System	Agilent	Cat# G2991AA
High Sensitivity D5000 Screentape	Agilent	Cat# 5067-5592
High Sensitivity D5000 Reagents	Agilent	Cat# 5067-5593

NextSeq 500550 Mid Output Kit v2.5 300 cycle kit	Illumina	Cat# 20024905
**Deposited Data**		
CVR-GLA-1	This paper	Genbank accession: MT882022
CVR-GLA-2	This paper	Genbank accession MT906650
CVR-GLA-3	This paper	Genbank accession MT906649
GenBank BioProject for SARS-CoV-2 clinical isolate and reverse genetics plasmid sequencing and genome assembly	This paper	GenBank BioProject PRJNA658321
**Cell Lines**		
Human: A549 cells	Laboratory of Benjamin Hale	
C. aethiops: Vero E6 cells	Laboratory of Michele Bouloy	
M. auratus: BHK-21 cells	ATCC	CCL-10
Caco-2	CVR Cytology Cell Bank	
Calu-3	ATCC	HTB-55
**Oligonucleotides**		
AW177-BamHI-SfiI-RFP-F': 5'-CTCTCGGATCCGGCCGAGAGGGCCATGAGCGAGCTGATTAAG-3'	This paper	N/A
AW178-EcoRI-SfiI-RFP-R': 5'-CTCTCGAATTCGGCCAGAGAGGCCTCACTTGTGCCCCAG-3'	This paper	N/A
MT_313:' HsTMPRSS2 5' SfiI: 5'-CTCTCTGGCCGAGAGGGCCATGGCTTTGAACTCAGGGTCACCAC-3'	This paper	N/A
MT_314: ' Hs TMPRSS2 3' SfiI: 5'-TCTCTCGGCCAGAGAGGCCTTAGCCGTCTGCCCTCATTTGTCG-3'	This paper	N/A
NEBNext Multiplex Oligos for Illumina 96 Unique Dual Index Primer Pairs Set 2	New England Biolabs	Cat# E6442S
**Recombinant DNA**		
pCCI-4K-SARS-CoV-2-Wuhan-Hu-1	This paper	GenBank accession: MT926410
pCCI-4K-SARS-CoV-2-mCherry	This paper	GenBank accession: MT926411
pCCI-4K-SARS-CoV-2-NanoLuc	This paper	GenBank accession: MT926412
pCCI-4K-SARS-CoV-2-ZsGreen	This paper	GenBank accession: MW289908
Homo sapiens ACE2 cDNA	Eurofins	GenBank accession: NM_001371415.1
Homo sapiens TMPRSS2 cDNA	Integrated DNA Technologies	GenBank accession: NM_005656
**Algorithms and Software**		

Graphpad Prism 8	https://www.graphpad.com/scientific-software/prism/	
Zeiss Zen	https://www.zeiss.com/microscopy/int/products/microscope-software/zen.html	
Burrows-Wheeler Aligner v0.7.5a & v0.7.17-r1188	http://bio-bwa.sourceforge.net/	
Tanoti	http://www.bioinformatics.cvr.ac.uk/tanoti.php	
ImageJ-1.53a	https://imagej.nih.gov	
Imaris Bitplane 9.0.2	https://imaris.oxinst.com/	
GIMP-2.10	https://www.gimp.org	
Biorender	biorender.com	
**Other Reagents**		
FastDigest restriction enzymes (Kfll; PacI; MluI; Eco81I; BamHI)	Thermo Fisher Scientific	Cat #FD2164; #FD2204; #FD0564; #FD0374; #FD0054
Tryptic Soy Agar	Becton Dickinson	Cat# BA-256665.03
Tryptic Soy Broth	Becton Dickinson	Cat# BA-257107.03
FastAP Thermosensitive Alkaline Phosphatase	Thermo Fisher Scientific	Cat# EF0651
Zymoclean Gel DNA Recovery Kit	Zymo Research	Cat# D4008
Genomic DNA Clean & Concentrator 10	Zymo Research	Cat# D4011
Invitrogen T4 DNA ligase	Thermo Fisher Scientific	Cat# 15224025
NucleoBond Xtra Midi EF	Machery Nagel	Cat# 740420.50
