# Supplementary material for: A plasmid DNA-launched SARS-CoV-2 reverse genetics system and coronavirus toolkit for COVID-19 research
Source: PLoS Biol. 2021 Feb 25;19(2):e3001091. doi: 10.1371/journal.pbio.3001091 (PMC7906417; doi:10.1371/journal.pbio.3001091)
Supplement: S1 Raw Images — (PDF) [file pbio.3001091.s011.pdf]

Figure 2D
ORF3a

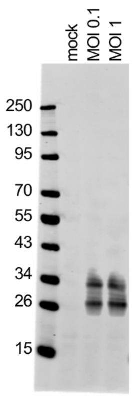

Figure 2D
N

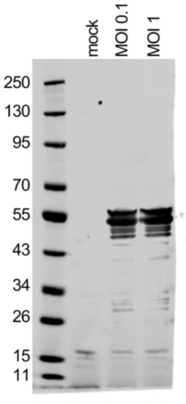

Figure 2D
nsp2

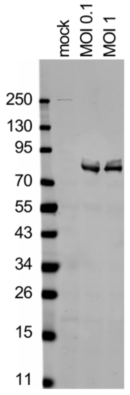

Figure 2D
β-actin

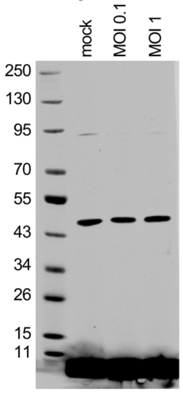

Figure 2E
ORF3a

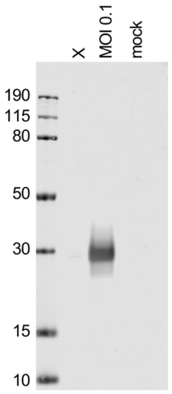

Figure 2E
N

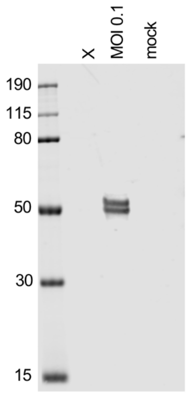

Figure 2E
nsp2

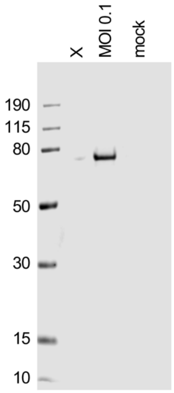

Figure 2G
ORF3a

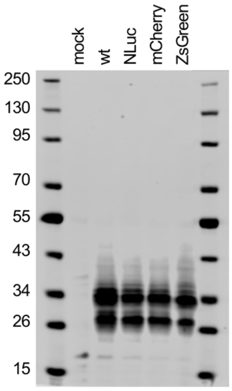

Figure 2G
N

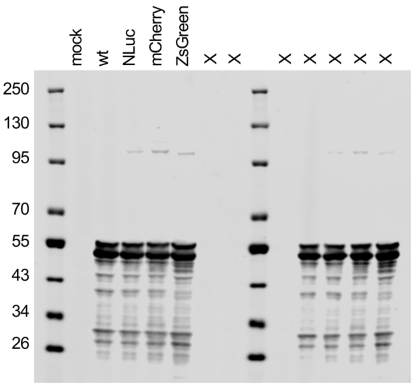

Figure 2G
nsp2

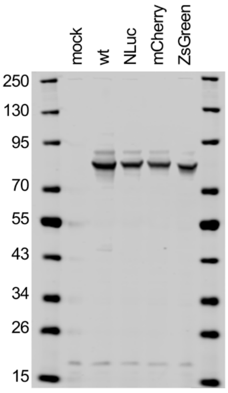

Figure 2G
actin

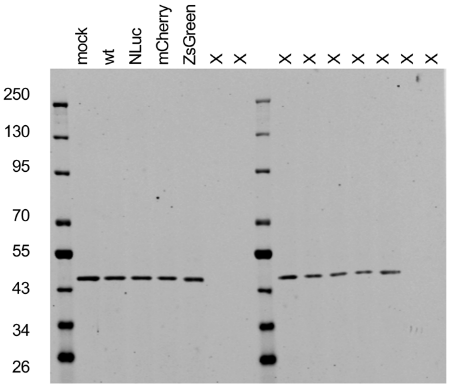

Figure 3A

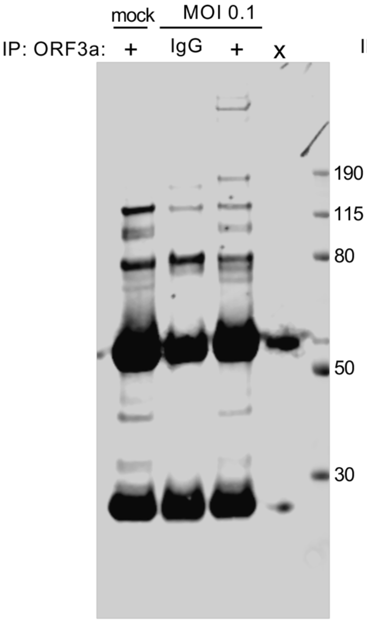

Figure 3A

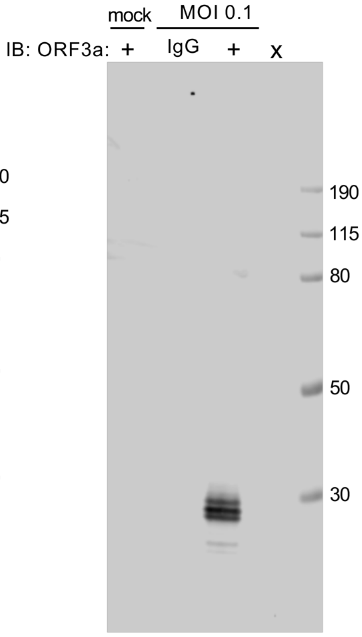

Figure 3B

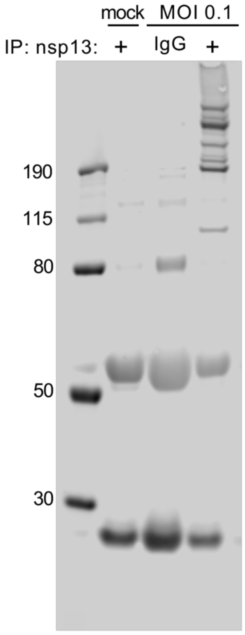

Figure 3B

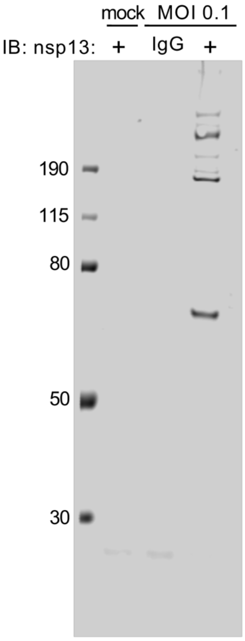

Figure 3C

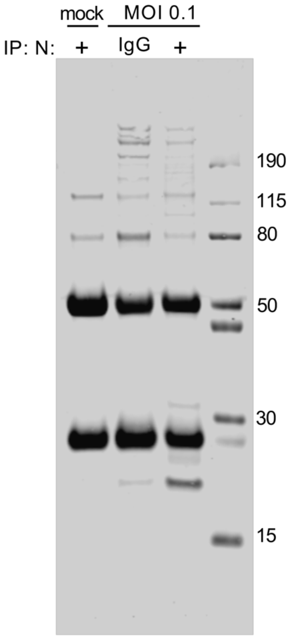

Figure 3C

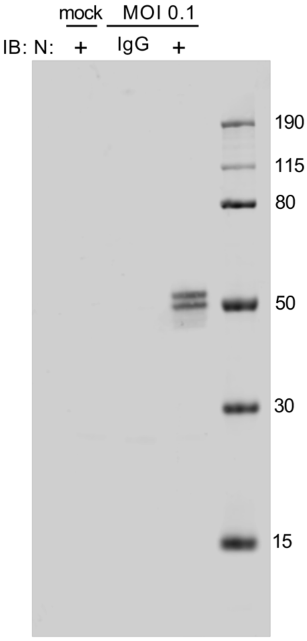

Figure 4B

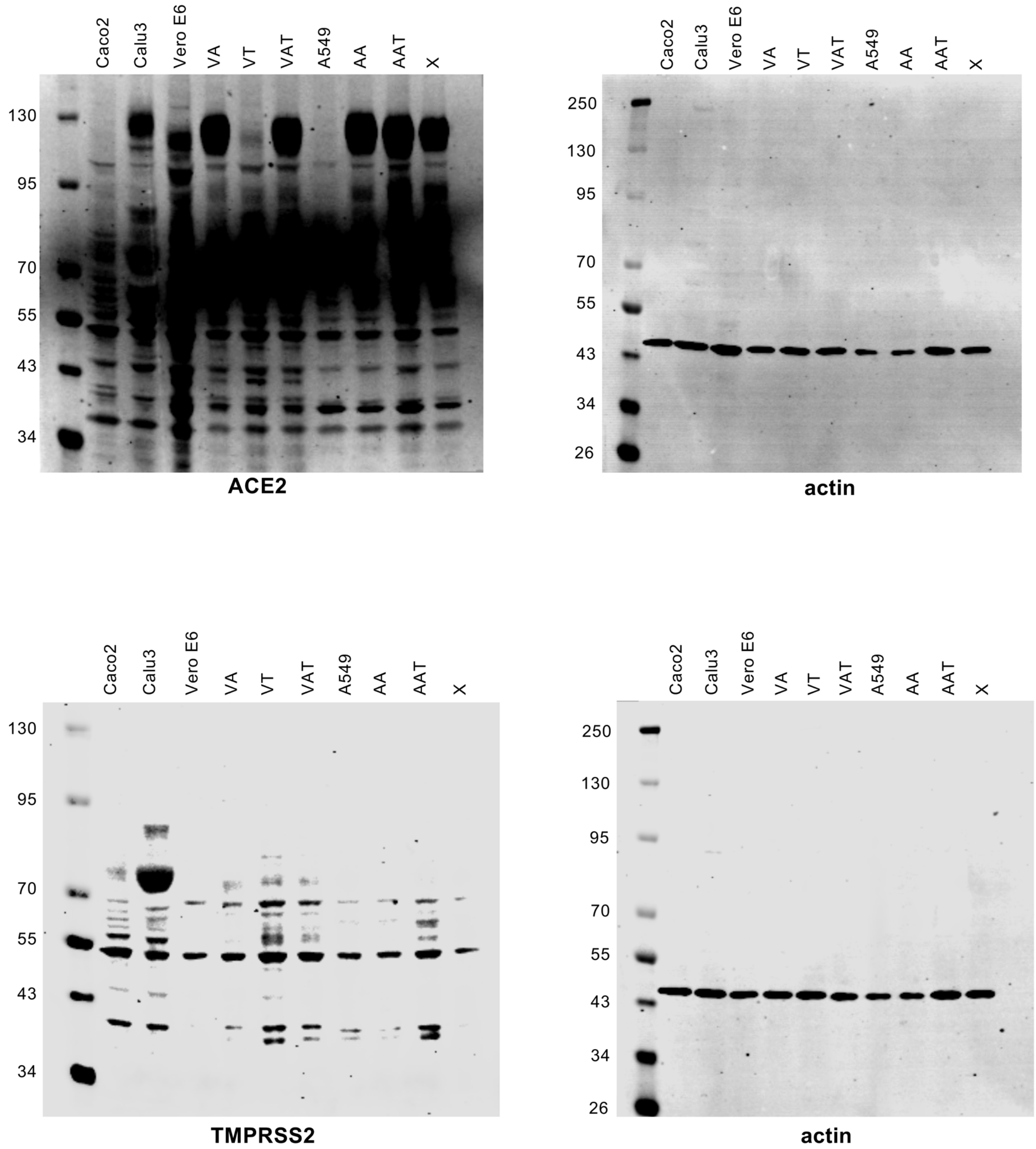
Caco2
Calu3
Vero E6
VA
VT
VAT
A549
AA
AAT
X
130
95
70
55
43
34
ACE2
250
130
95
70
55
43
34
26
actin
TMPRSS2
actin

S3 Fig. 1 of 2

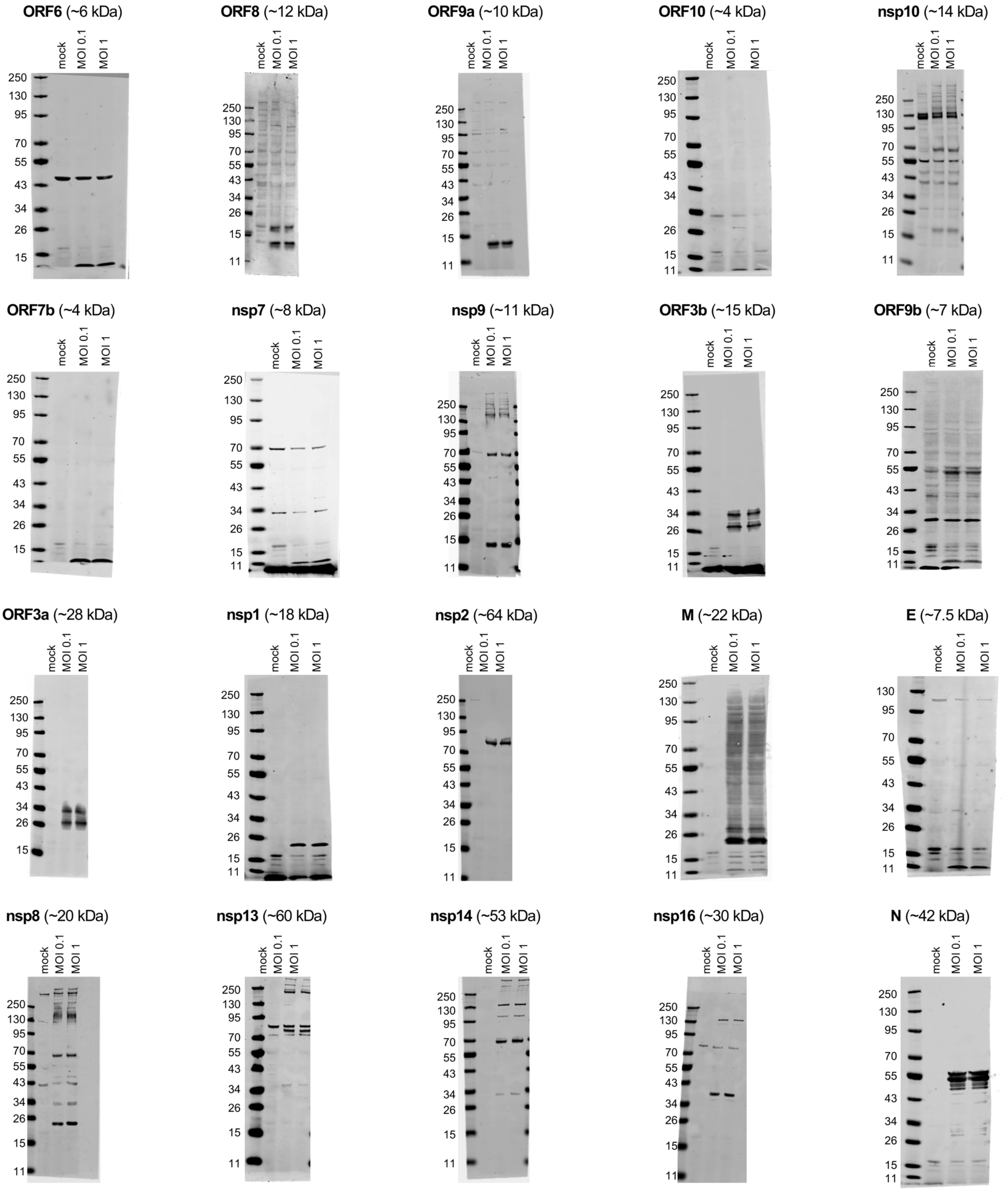
ORF6 (~6 kDa)
ORF8 (~12 kDa)
ORF9a (~10 kDa)
ORF10 (~4 kDa)
nsp10 (~14 kDa)
ORF7b (~4 kDa)
nsp7 (~8 kDa)
nsp9 (~11 kDa)
ORF3b (~15 kDa)
ORF9b (~7 kDa)
ORF3a (~28 kDa)
nsp1 (~18 kDa)
nsp2 (~64 kDa)
M (~22 kDa)
E (~7.5 kDa)
nsp8 (~20 kDa)
nsp13 (~60 kDa)
nsp14 (~53 kDa)
nsp16 (~30 kDa)
N (~42 kDa)
mock
MOI 0.1
MOI 1
250
130
95
70
55
43
34
26
15
11

S3 Fig. 2 of 2

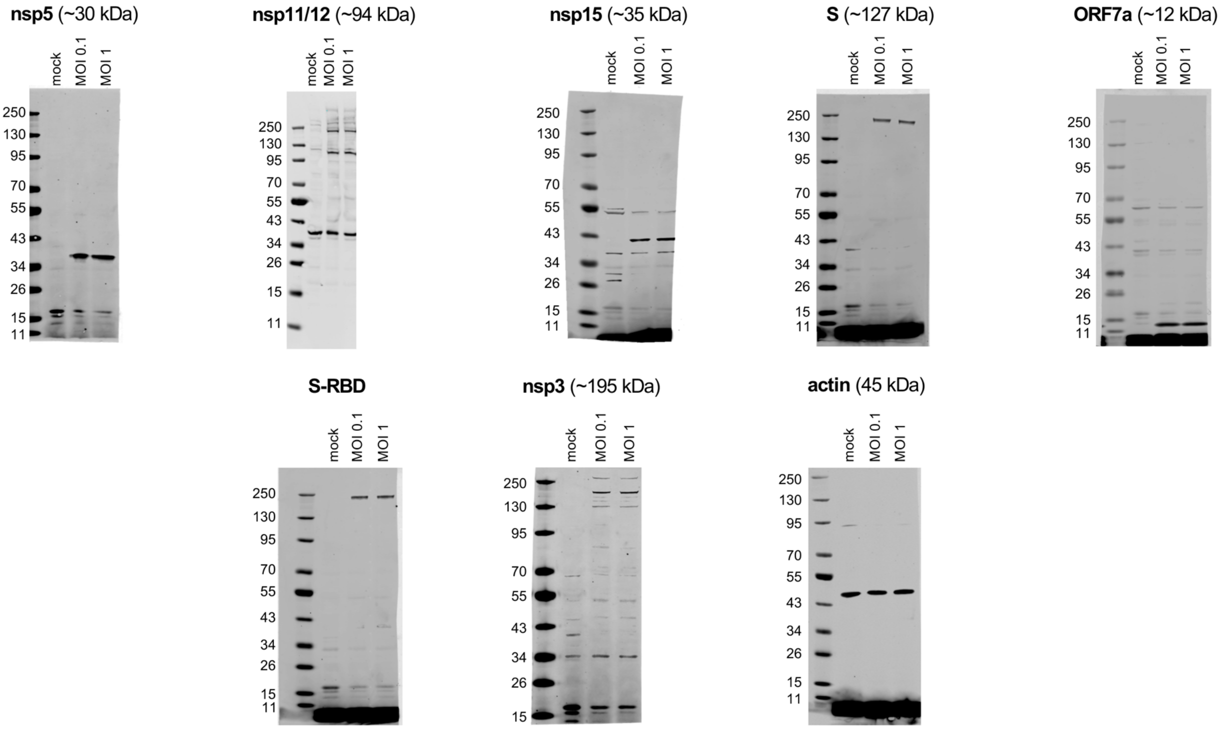

nsp5 (~30 kDa)
mock
MOI 0.1
MOI 1
250
130
95
70
55
43
34
26
15
11
nsp11/12 (~94 kDa)
mock
MOI 0.1
MOI 1
250
130
95
70
55
43
34
26
15
11
nsp15 (~35 kDa)
mock
MOI 0.1
MOI 1
250
130
95
70
55
43
34
26
15
11
S (~127 kDa)
mock
MOI 0.1
MOI 1
250
130
95
70
55
43
34
26
15
11
ORF7a (~12 kDa)
mock
MOI 0.1
MOI 1
250
130
95
70
55
43
34
26
15
11
S-RBD
mock
MOI 0.1
MOI 1
250
130
95
70
55
43
34
26
15
11
nsp3 (~195 kDa)
mock
MOI 0.1
MOI 1
250
130
95
70
55
43
34
26
15
actin (45 kDa)
mock
MOI 0.1
MOI 1
250
130
95
70
55
43
34
26
15
11

S4 Fig.

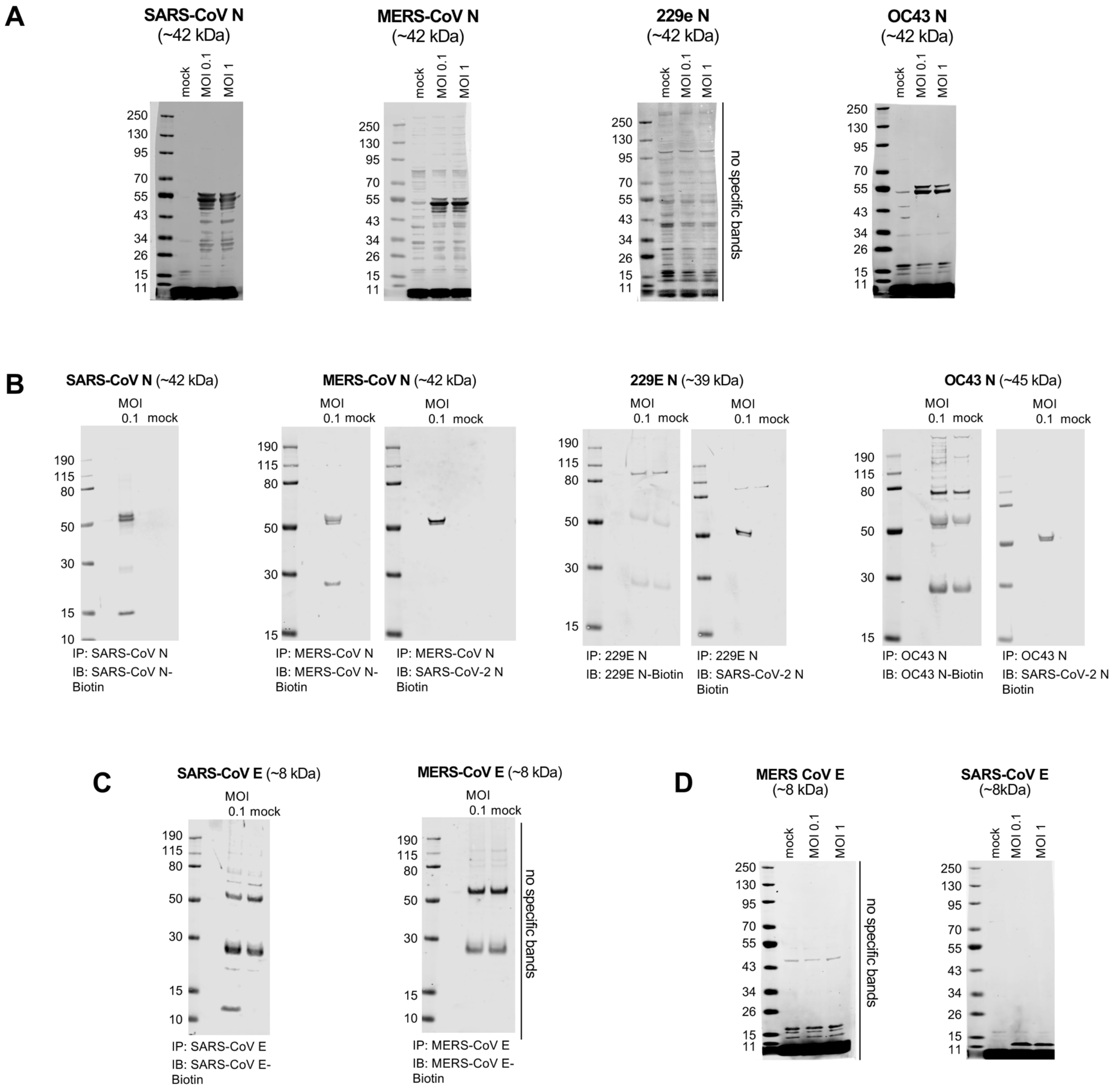

A
SARS-CoV N
(~42 kDa)
mock
MOI 0.1
MOI 1
250
130
95
70
55
43
34
26
15
11
MERS-CoV N
(~42 kDa)
229e N
(~42 kDa)
no specific bands
OC43 N
(~42 kDa)
B
SARS-CoV N (~42 kDa)
MOI
0.1 mock
190
115
80
50
30
15
10
IP: SARS-CoV N
IB: SARS-CoV N-Biotin
MERS-CoV N (~42 kDa)
IP: MERS-CoV N
IB: MERS-CoV N-Biotin
IP: MERS-CoV N
IB: SARS-CoV-2 N Biotin
229E N (~39 kDa)
IP: 229E N
IB: 229E N-Biotin
IP: 229E N
IB: SARS-CoV-2 N Biotin
OC43 N (~45 kDa)
IP: OC43 N
IB: OC43 N-Biotin
IP: OC43 N
IB: SARS-CoV-2 N Biotin
C
SARS-CoV E (~8 kDa)
IP: SARS-CoV E
IB: SARS-CoV E-Biotin
MERS-CoV E (~8 kDa)
no specific bands
IP: MERS-CoV E
IB: MERS-CoV E-Biotin
D
MERS CoV E
(~8 kDa)
no specific bands
SARS-CoV E
(~8kDa)

S5 Fig. 1 of 2

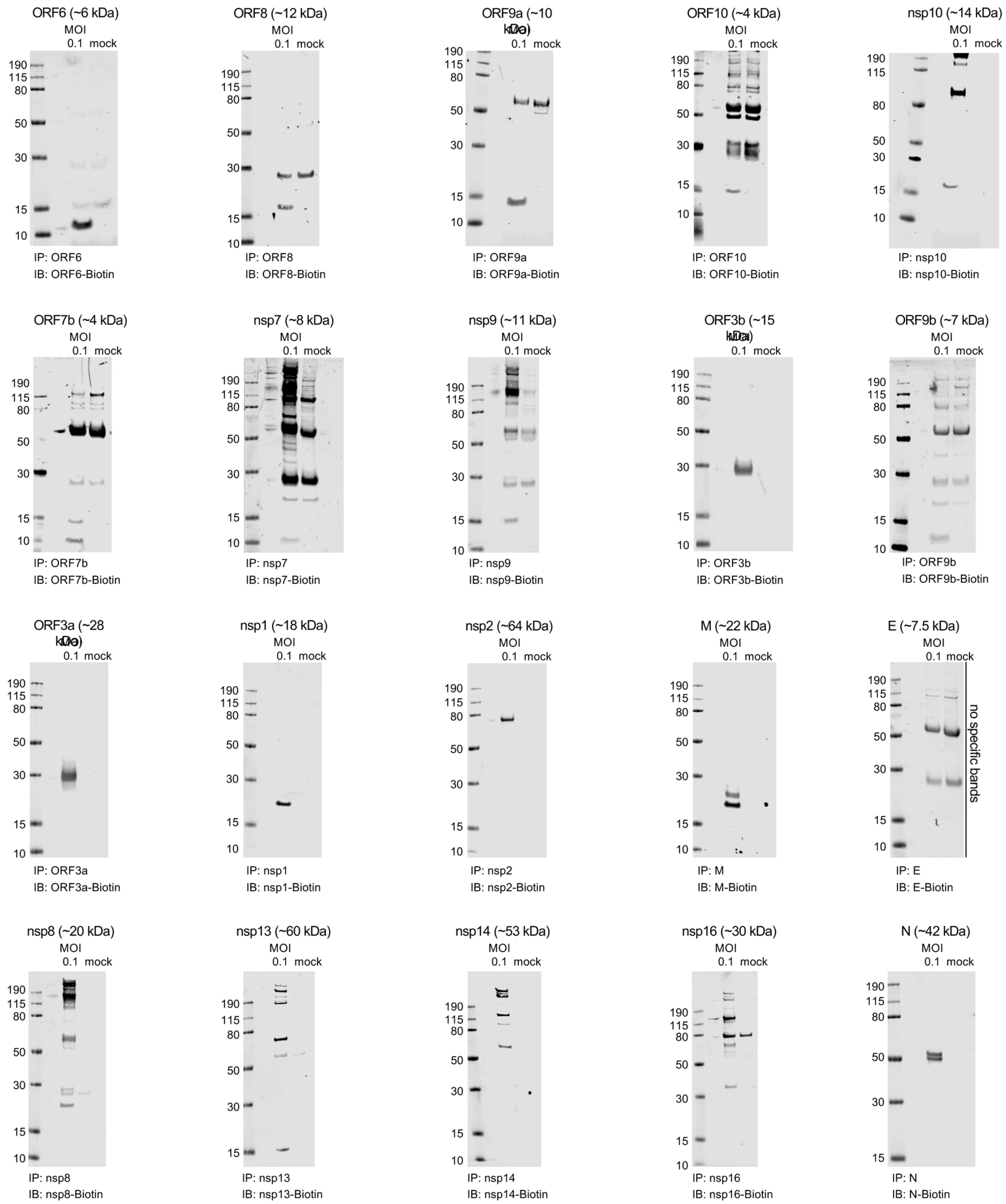
ORF6 (~6 kDa)
MOI
0.1 mock
IP: ORF6
IB: ORF6-Biotin
ORF8 (~12 kDa)
MOI
0.1 mock
IP: ORF8
IB: ORF8-Biotin
ORF9a (~10 kDa)
0.1 mock
IP: ORF9a
IB: ORF9a-Biotin
ORF10 (~4 kDa)
MOI
0.1 mock
IP: ORF10
IB: ORF10-Biotin
nsp10 (~14 kDa)
MOI
0.1 mock
IP: nsp10
IB: nsp10-Biotin
ORF7b (~4 kDa)
MOI
0.1 mock
IP: ORF7b
IB: ORF7b-Biotin
nsp7 (~8 kDa)
MOI
0.1 mock
IP: nsp7
IB: nsp7-Biotin
nsp9 (~11 kDa)
MOI
0.1 mock
IP: nsp9
IB: nsp9-Biotin
ORF3b (~15 kDa)
0.1 mock
IP: ORF3b
IB: ORF3b-Biotin
ORF9b (~7 kDa)
MOI
0.1 mock
IP: ORF9b
IB: ORF9b-Biotin
ORF3a (~28 kDa)
0.1 mock
IP: ORF3a
IB: ORF3a-Biotin
nsp1 (~18 kDa)
MOI
0.1 mock
IP: nsp1
IB: nsp1-Biotin
nsp2 (~64 kDa)
MOI
0.1 mock
IP: nsp2
IB: nsp2-Biotin
M (~22 kDa)
MOI
0.1 mock
IP: M
IB: M-Biotin
E (~7.5 kDa)
MOI
0.1 mock
no specific bands
IP: E
IB: E-Biotin
nsp8 (~20 kDa)
MOI
0.1 mock
IP: nsp8
IB: nsp8-Biotin
nsp13 (~60 kDa)
MOI
0.1 mock
IP: nsp13
IB: nsp13-Biotin
nsp14 (~53 kDa)
MOI
0.1 mock
IP: nsp14
IB: nsp14-Biotin
nsp16 (~30 kDa)
MOI
0.1 mock
IP: nsp16
IB: nsp16-Biotin
N (~42 kDa)
MOI
0.1 mock
IP: N
IB: N-Biotin

S5 Fig. 2 of 2

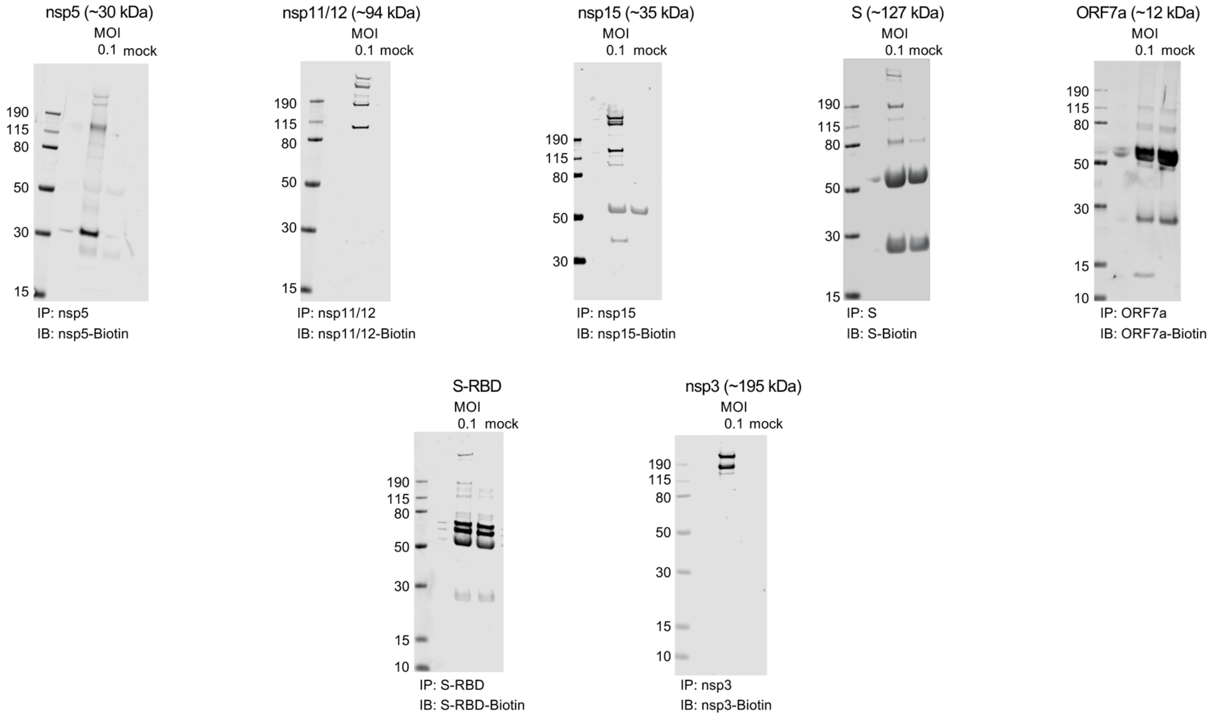

nsp5 (~30 kDa)
MOI
0.1 mock
190
115
80
50
30
15
IP: nsp5
IB: nsp5-Biotin
nsp11/12 (~94 kDa)
MOI
0.1 mock
190
115
80
50
30
15
IP: nsp11/12
IB: nsp11/12-Biotin
nsp15 (~35 kDa)
MOI
0.1 mock
190
115
80
50
30
IP: nsp15
IB: nsp15-Biotin
S (~127 kDa)
MOI
0.1 mock
190
115
80
50
30
15
IP: S
IB: S-Biotin
ORF7a (~12 kDa)
MOI
0.1 mock
190
115
80
50
30
15
10
IP: ORF7a
IB: ORF7a-Biotin
S-RBD
MOI
0.1 mock
190
115
80
50
30
15
10
IP: S-RBD
IB: S-RBD-Biotin
nsp3 (~195 kDa)
MOI
0.1 mock
190
115
80
50
30
15
10
IP: nsp3
IB: nsp3-Biotin
